# Single-cell metabolic fingerprints discover a cluster of circulating tumor cells with distinct metastatic potential

**DOI:** 10.1038/s41467-023-38009-3

**Published:** 2023-04-29

**Authors:** Wenjun Zhang, Feifei Xu, Jiang Yao, Changfei Mao, Mingchen Zhu, Moting Qian, Jun Hu, Huilin Zhong, Junsheng Zhou, Xiaoyu Shi, Yun Chen

**Affiliations:** 1 School of Pharmacy, Nanjing Medical University, Nanjing, 211166 China; 2 Department of General Surgery, Jiangsu Cancer Hospital (Jiangsu Institute of Cancer Research, Nanjing Medical University Affiliated Cancer Hospital), Nanjing, 210009 China; 3 Department of Clinical Laboratory, Jiangsu Cancer Hospital (Jiangsu Institute of Cancer Research, Nanjing Medical University Affiliated Cancer Hospital), Nanjing, 210009 China; 4 School of Computer Science and Technology, Nanjing Normal University, Nanjing, 210046 China; 5 State Key Laboratory of Reproductive Medicine, Nanjing, 211166 China; 6 Key Laboratory of Cardiovascular and Cerebrovascular Medicine, Nanjing, 211166 China

**Keywords:** Mass spectrometry, Predictive markers

## Abstract

Circulating tumor cells (CTCs) are recognized as direct seeds of metastasis. However, CTC count may not be the “best” indicator of metastatic risk because their heterogeneity is generally neglected. In this study, we develop a molecular typing system to predict colorectal cancer metastasis potential based on the metabolic fingerprints of single CTCs. After identification of the metabolites potentially related to metastasis using mass spectrometry-based untargeted metabolomics, setup of a home-built single-cell quantitative mass spectrometric platform for target metabolite analysis in individual CTCs and use of a machine learning method composed of non-negative matrix factorization and logistic regression, CTCs are divided into two subgroups, C1 and C2, based on a 4-metabolite fingerprint. Both in vitro and in vivo experiments demonstrate that CTC count in C2 subgroup is closely associated with metastasis incidence. This is an interesting report on the presence of a specific population of CTCs with distinct metastatic potential at the single-cell metabolite level.

## Abstract (web summary)

Circulating tumor cells (CTCs) are the tumor cells responsible for metastasis. Here, the authors develop a home-built single-cell quantitative mass spectrometric platform and discover a cluster of CTCs with distinct metastatic potential using a molecular typing system based on the single-cell metabolic fingerprints.

## Introduction

Cancer has emerged as a global public health issue and is a leading cause of death^1^. For most cancer patients, it is not the primary lesion that causes death but metastasis^2^. Among various cancers, colorectal cancer has a high incidence rate of metastasis. Nearly half of colorectal cancer patients develop metastasis^3^, which accounts for as many as 90% of colorectal cancer-related deaths^4^.

To date, much evidence has shown that early and accurate detection of cancer metastasis is critical for the prognosis and treatment of colorectal cancer patients^5^. Conventional methods such as magnetic resonance imaging (MRI) and computed tomography (CT) have been widely used to detect the incidence of metastasis. However, these methods typically can only identify metastatic lesions larger than 75 μm^6^, hence easily overlooking small lesions and playing only a small role in early warning and predicting cancer metastasis the first time. In fact, cancer metastasis is a multistep process that involves local cancer cell acquisition of migratory potential, followed by cell expansive growth and migration, and eventually cancer-derived material transportation around the body and arrest at distant sites^7^.

Among the released cancer-derived materials, a proportion of circulating tumor cells (CTCs) have the capability to metastasize^8,9^. CTCs are a small group of malignant cells that are shed from primary lesions and carried in the bloodstream^7^. To date, the number of CTCs has been recommended as an indicator of metastasis in clinical practice guidelines and recommendations for some cancers^10,11^. For example, the cutoff value of CTC count for unfavorable prognosis of metastatic colorectal cancer is ≥3 CTCs/7.5 ml. However, increasing evidence has shown no significant difference in metastasis status and clinical outcome between patients with higher and lower CTC counts. Thus, the number of CTCs may not be the “best” indicator of metastatic risk^12^. Further studies revealed that cancer metastasis is actually initiated by a subpopulation of CTCs, but not bulk CTCs^12,13^. Specifically, only certain CTCs in the population are capable of metastasis^10^. Therefore, identification of a CTC subgroup with metastatic potential may be more effective than superficial CTC bulk counting in metastasis prediction and prognosis evaluation of colorectal cancer.

The metastatic potential of CTCs is closely related to a variety of factors, among which their capability to overcome extraneous variables in the bloodstream is an important one^14^. This factor is largely supported by metabolic reprogramming, a hallmark of metastatic cancer cells^15^. During metastasis, CTCs could reprogram their metabolism and have an altered metabolic phenotype^16^. The difference in CTC metastatic potential may result from the heterogeneity in their metabolic phenotype^13,17^. Thus, metabolic profiling of individual CTCs and characterization of the metabolic fingerprint relative to cancer metastasis may help distinguish CTCs with metastatic potential and build a molecular typing system of colorectal cancer at the single-CTC metabolite level.

Currently, mass spectrometry-based metabolomic analysis has been broadly applied to identify metabolomic abnormalities. With the goal of studying the heterogeneity within cells, single-cell mass spectrometry has emerged to profile metabolites at single-cell resolution^18,19^. However, quantitative information on target metabolites at the single-cell level is usually missing in current studies, whereas quantification is necessary for monitoring the role of specific CTCs metabolic phenotype with respect to cell heterogeneity and is normally performed for marker development and therapeutic target validation in clinical practice^20^. Several commonly acknowledged challenges exist in single-cell quantitative mass spectrometry^21^, including (1) controlled extraction of subpicoliter material from the cell, (2) adjustment of nonbiological variations associated with the experimental process, and (3) enhancement of repeatability and sensitivity.

**Fig. 1:**
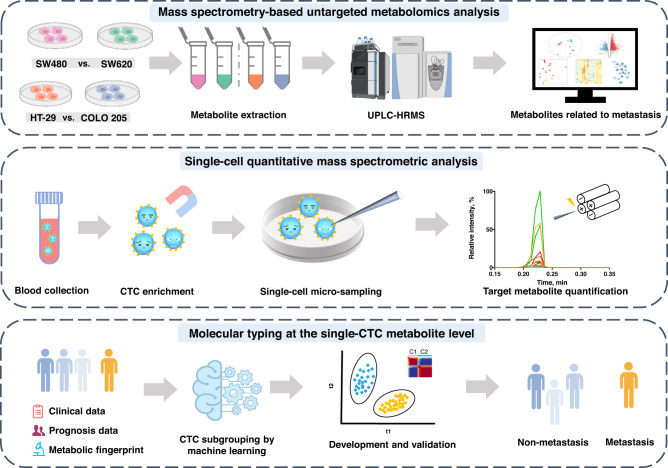
Schematic workflow of circulating tumor cell (CTC) subgrouping and colorectal cancer metastasis prediction using home-built single-cell quantitative mass spectrometry platform. First, two pairs of colorectal cell lines with differential metastatic potential were screened using mass spectrometry-based untargeted metabolomics, and metabolites with differential abundance correlating with cancer metastatic risk were identified. Then, CTCs were isolated from colorectal cancer patients. Cellular content was extracted from single CTCs, and target metabolites were analyzed using single-cell quantitative mass spectrometry. A molecular typing system of colorectal cancer was developed to identify metabolic fingerprint correlated with metastasis and to construct a CTC subgrouping approach to predict the incidence of metastasis in colorectal cancer patients at the single-cell metabolite level. Some graphical elements were created with BioRender.com (accessed on 24 March 2023). UPLC-HRMS ultra-performance liquid chromatography-tandem high-resolution mass spectrometry.

In this study, a molecular typing system of colorectal cancer was built to predict the incidence of cancer metastasis based on the metabolic fingerprint of CTCs using a home-built single-cell quantitative mass spectrometry platform (Fig. 1 and Supplementary Fig. 1). First, a panel of metabolites with differential abundance was identified by metabolic screening of two pairs of human colorectal cancer cell lines with different metastatic abilities. Afterward, a single-cell quantitative mass spectrometric analysis was developed to measure target metabolites in individual CTCs from enrolled colorectal cancer patients with no metastasis. Then, the CTCs were classified based on the proposed metabolic fingerprint, and the CTC subgroup associated with cancer metastatic potential was discerned using both in vitro and in vivo functional assays. This developed molecular typing system was further tested in a test cohort and an independent prospective cohort.

## Results

### Metabolic profiling of low- and high-metastatic human colorectal cancer cell lines

**Fig. 2:**
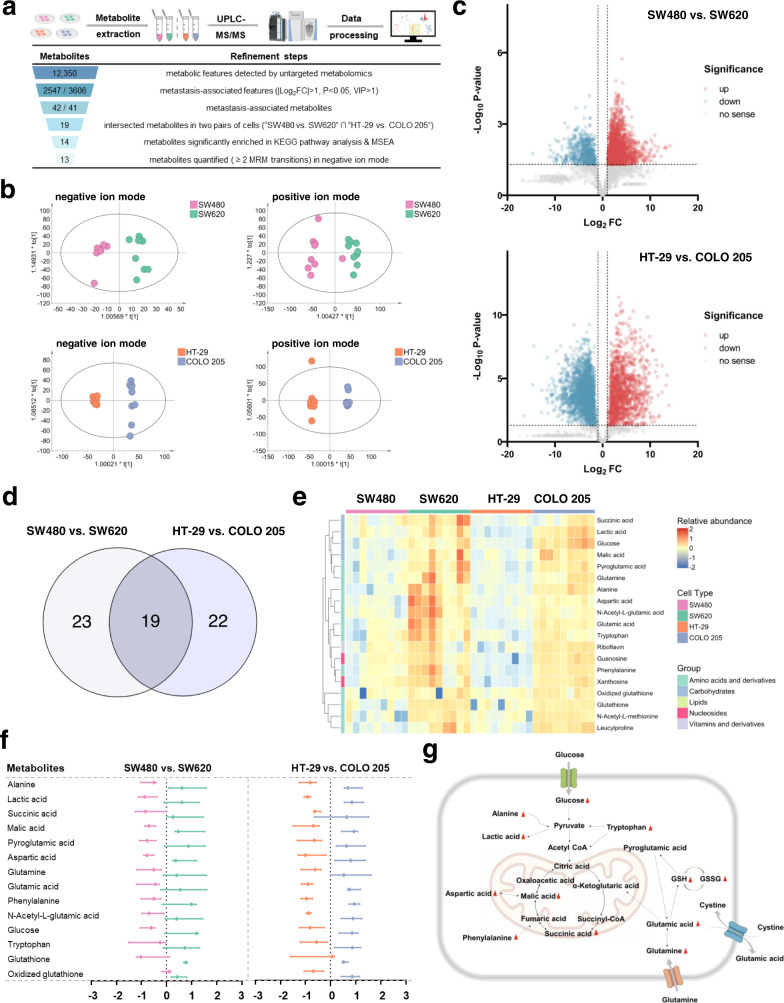
Metabolic profiling of colorectal cell lines with differential metastatic potential by mass spectrometry-based untargeted metabolomic analysis. **a** Schematic pipeline of metabolic screening (upper) and steps for refining the potential metabolites (lower). **b** OPLS-DA plots of individual cells of SW480/SW620 and HT-29/COLO 205 in negative and positive ion modes (R2X(cum) = 0.705, R2Y(cum) = 0.952, Q2(cum) = 0.725 and R2X(cum) = 0.679, R2Y(cum) = 0.994, Q2(cum) = 0.987 in negative ion mode for SW480/SW620 and HT-29/COLO 205; R2X(cum) = 0.426, R2Y(cum) = 0.869, Q2(cum) = 0.785 and R2X(cum) = 0.632, R2Y(cum) = 0.996, Q2(cum) = 0.961 in positive ion mode for SW480/SW620 and HT-29/COLO 205). **c** Volcano plots of metabolite abundance in SW480/SW620 cells and HT-29/COLO 205 cells. The red and cyan dots represent significantly increased/decreased metabolic features (|Log_2_FC | >1, *p* < 0.05, VIP > 1). **d** The intersection of the metabolites with significantly differential abundance obtained from the two pairs of cell lines (SW480 vs. SW620, HT-29 vs. COLO 205). **e** Heatmap of the relative abundance (Log_10_ transformation) of the shared metabolites with differential abundance in the two pairs of cells (*n* = 9). **f** *Z*-score plot of 14 representative metabolites with differential abundance in SW480/SW620 cells and HT-29/COLO 205 cells (*n* = 9 independent experiments). Data are presented as median with interquartile range and points are colored by assigned cell type. **g** Metabolic network of representative altered metabolites. Some graphical elements in (**a**) and (**g**) were created with BioRender.com (accessed on 24 March 2023). UPLC-HRMS ultra-performance liquid chromatography-tandem high-resolution mass spectrometry, FC fold change, VIP variable important in projection, KEGG Kyoto Encyclopedia of Genes and Genomes, MSEA metabolite set enrichment analysis, MRM multiple reaction monitoring.

Mass spectrometry-based untargeted metabolomic analysis was performed in two pairs of colorectal cancer cell lines with different metastatic abilities (Fig. 2a). Each pair of cell lines was comprised of primary and metastatic colorectal cancer cell lines (SW480 vs. SW620; HT-29 vs. COLO 205)^22^. After quality control, data filtering, and removal of missing values, a total of 12,350 metabolic features, including 8721 in positive ion mode and 3629 in negative ion mode, were detected and used for the subsequent principal component analysis (PCA). The PCA plots in Supplementary Fig. 3a show a clear clustering of cell lines, confirming the suitability of using metabolic features to distinguish phenotypically different cells. Orthogonal partial least-squares discriminant analysis (OPLS-DA) analysis was further conducted in the two pairs of cell lines, and the cross-validated Q2 (cum) values were 0.725 (negative ion mode), 0.785 (positive ion mode) for SW480 and SW620 cells and 0.987 (negative ion mode), 0.961 (positive ion mode) for HT-29 and COLO 205 cells, respectively (Fig. 2b), indicating the good predictive ability of metabolic features. Significant changes in the level of 2547 (SW480 vs. SW620) and 3606 (HT-29 vs. COLO 205) metabolic features (|Log_2_FC|> 1, *p* < 0.05, VIP > 1) were observed, and these features were used for subsequent analysis (Fig. 2c).

### Identification of metabolites indicative of metastasis potential

Based on the metabolic features found in the cell dataset, the corresponding metabolites were identified based on their retention time, accurate mass and MS/MS spectra information. As a result, we annotated these metabolic features to 42 and 41 metabolite molecules (SW480 vs. SW620 and HT-29 vs. COLO 205), respectively (Supplementary Table 1 and Source Data). The metabolites with differential abundance are shown in Supplementary Fig. 3b. Among these metabolites, 19 metabolites were shared by the two pairs of cell lines (Fig. 2d). Interestingly, these metabolites exhibited increased levels in the metastatic cell lines (i.e., SW620 and COLO 205 cells) compared to their counterparts (Fig. 2e).

Subsequently, we conducted Kyoto Encyclopedia of Genes and Genomes (KEGG) pathway analysis to identify the most strongly influenced metabolic pathways using the identified metabolites. The results indicated that these metabolites were significantly enriched in six metabolic pathways, centering on amino acid metabolism and glutathione metabolism (Supplementary Fig. 3c). To further explore the functional differences of these metabolites, we also performed metabolite set enrichment analysis (MSEA). As shown in Supplementary Fig. 3d, the categories with significant enrichment were related to metabolic pathways involving glutathione metabolism, amino acid metabolism and the Warburg effect. As tools of knowledge-based dimensionality reduction, these pathway analyses are often beneficial to exclude numerous small pathways that are redundant with larger pathways and complicate interpretation^23^. Our results also provided some evidence that dimensionality reduction did not oversimplify the dataset here (Supplementary Fig. 4). Altogether, a total of 14 metabolites in these pathways were screened out. Their relative abundance (*z*-score) in SW480/SW620 and HT-29/COLO 205 cell lines and representative metabolic networks are shown in Fig. 2f, g. Correlation analysis between these metabolites is also shown in Supplementary Fig. 3e. Owing to the better coverage of these selected metabolites in negative ion mode, negative ion mode was preferable for cancer molecular typing in this study, and 14 metabolites were subjected to single CTC analysis.

### Development of a home-built single-cell quantitative mass spectrometry platform

In this work, a home-built single-cell quantitative mass spectrometric platform was developed to monitor the 14 identified metabolites in individual CTCs from enrolled colorectal cancer patients. During method development, three pivotal challenges in single-cell analysis as described earlier should be carefully addressed.

**Fig. 3:**
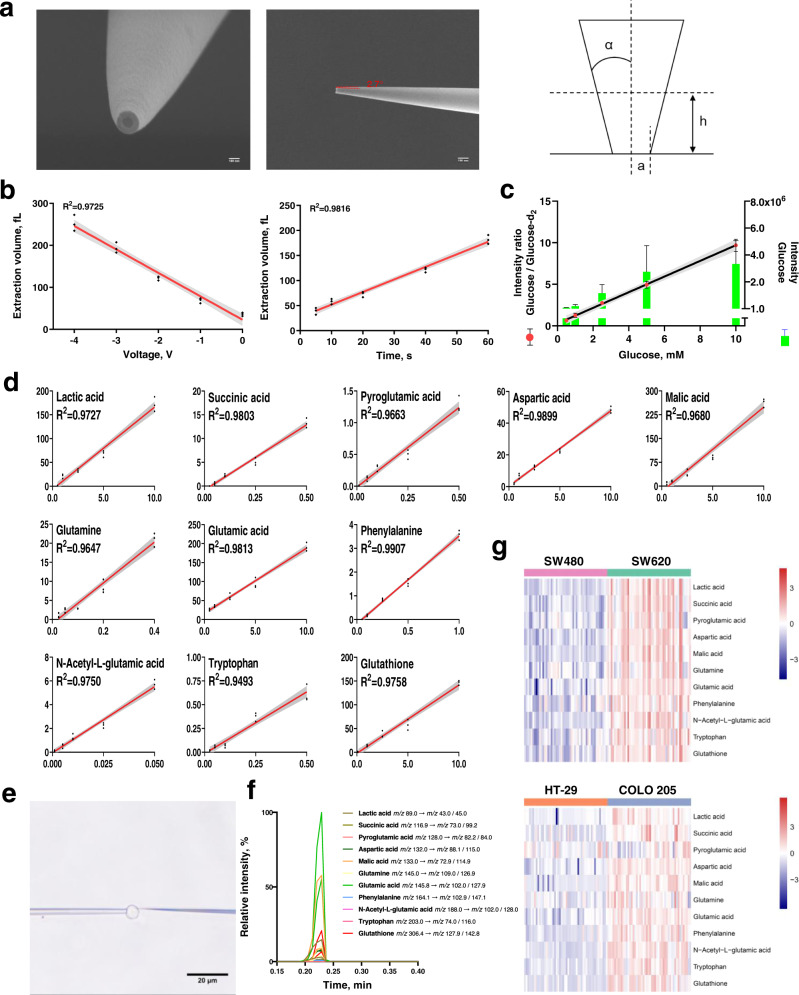
Development of a home-built single-cell quantitative mass spectrometry platform. **a** SEM image of the nanocapillary tip (left: front view, middle: side view, right: model of the tip for volume estimation). The data are representative of six independent experiments with similar results. **b** Dependence of the extracted volume on extraction time at −2 V (left) and extraction voltage in 40 s (right). The shaded error bands indicate 95% confidence intervals. **c** Mass response of target metabolite in single-cell quantitative mass spectrometry. After extracting 1 mM glucose-d_2_ as the internal standard and glucose at a concentration in the range of 0.5–10 mM, the mass response of glucose did not increase proportionally in the concentration, while a linear dependence of the intensity ratio of glucose and glucose-d_2_ on glucose concentration was observed (*n* = 3 replicates at each concentration). Data are plotted as mean ± SEM. The error bars of glucose intensity are presented over the histogram for clarity. The shaded error bands indicate 95% confidence intervals. **d** Calibration curves of 11 target metabolites. The curves were constructed by plotting MS signal ratio of metabolite and internal standard against concentration of calibration standards. The shaded error bands indicate 95% confidence intervals. **e** Bright-field image of a nanocapillary tip inserted into the cell and cellular extraction. Experiments were repeated six times with similar results. **f** A typical MRM chromatogram of target metabolites at the single-cell level. **g** Heatmaps of the relative abundance (Log_10_ transformation) of the target metabolites in single SW480/SW620 cells and HT-29/COLO 205 cells (*n* = 50).

Referring to the electro-osmotic extraction method reported by Mirkin’s group, we successfully filled subpicoliter volumes of cellular contents into nanocapillaries based on electro-osmotic flow^24^. The scanning electron microscopy (SEM) images of nanocapillary tips are shown in Fig. 3a and indicate a ∼100 nm opening. The filled volume V was estimated from the angle α between the cone element and its axis, the observed diameter a, and the height of the meniscus h formed between the hydrophobic electrolyte and the extracted aqueous phase using the formula $V=\frac{{{{{{\rm{\pi }}}}}}{a}^{3}}{3{{\tan }}\alpha }({\left(1+L{{\tan }}\alpha \right)}^{3}-1),\, {L}=h/a$. Theoretically, the value of volume V is mainly dependent on the voltage and the time applied to the electrodes. In agreement with this speculation, our results demonstrated a linear relationship of the extracted volume with the applied voltage and time. Specifically, the volume increases linearly with the applied voltage between 0 V and −4 V at a fixed time of 40 s (Fig. 3b) and increases in proportion to the time in the range of 5–60 s at a fixed voltage of −2 V (Fig. 3b). Correspondingly, the images of nanocapillary tip after extraction are shown in Supplementary Fig. 5. Ultimately, an extraction condition of −2 V for 40 s was chosen. The estimated volume extracted from the cell was ~120 fL.

To efficiently reduce nonbiological variations during single-cell analysis, we corrected technical variability using spike-in approach by normalizing the analyte signal to the signal of stable isotope-labeled internal standard^25^, and removed batch effects using Combat function^26^. A pre-experiment was performed by sequential extraction of glucose solution with a concentration of 0.5–10 mM and 1 mM glucose-d_2_ as the internal standard using the extraction conditions optimized above. As shown in Fig. 3c, the mass response of glucose did not increase proportionally to its concentration; glucose-d_2_ at the same concentration had an inconsistent response, confirming the presence of nonbiological variations. The corresponding MRM chromatograms are shown in Supplementary Fig. 6. After correction of the variations, a linear dependence of the glucose/glucose-d_2_ ratio *y* on the concentration of glucose *x* was observed (*y* = 0.9414*x* + 0.2833, *R*^2^ = 0.9718, Fig. 3c).

To improve the sensitivity and repeatability of the mass spectrometry platform, MRM transitions of 14 target metabolites were all optimized. Among these metabolites, alanine has only one reliable MRM transition in negative ion mode. Ultimately, 13 target metabolites were selected, and two MRM transitions with the best signal-to-noise (S/N) ratio were monitored for each metabolite. Their MRM transitions are summarized in Supplementary Table 2. Quantitative information of the target metabolites was obtained by integrating the areas in the MRM transitions of target metabolites and internal standard. Using 5% bovine serum albumin (BSA) as surrogate matrix, LOD (the limit of detection), LLOQ (the lower limit of quantification) and linear range of each metabolite were determined (Supplementary Table 3). Calibration curves of the target metabolites were obtained (Fig. 3d). We also evaluated accuracy and precision of the method by measuring the response of the target metabolites at LLOQ as quality control (Supplementary Table 3). Notably, the intraday variations ranged from 8.2 to 18.1% and the interday variations ranged from 8.2 to 17.5%, meeting the general validation criteria^27^. This result also confirmed that the nonbiological variations were well reduced and good reproducibility was achieved.

The optimized single-cell quantitative mass spectrometry method was then applied for cell analysis. Images of capillary insertion and cellular extraction are shown in Fig. 3e. The collected MRM chromatogram from a single cell is also indicated (Fig. 3f). We further determined the levels of 13 target metabolites in cultured cells. Among these metabolites, eleven of them had apparent MS signal. After sample size estimation, 50 cells were analyzed for each cell line (Fig. 3g). Large variations with relative standard deviation (RSD) of ~60.0% in the same type of cells were observed. These variations were much larger than the nonbiological variations, reflecting cell heterogeneity even in genetically identical cells, which may be caused by differences in the stage of cell cycle at which a cell was captured^28^. Moreover, the nonbiological variations were significantly lower than the variations in the two pairs of cells altogether (85.6% for SW480 and SW620 cells, 90.9% for HT-29 and COLO 205 cells), which were more representative of the cells in real samples. Therefore, it is reasonable to expect a larger variation in individual CTCs.

### Discovery of CTC metabolic subgroups

**Fig. 4:**
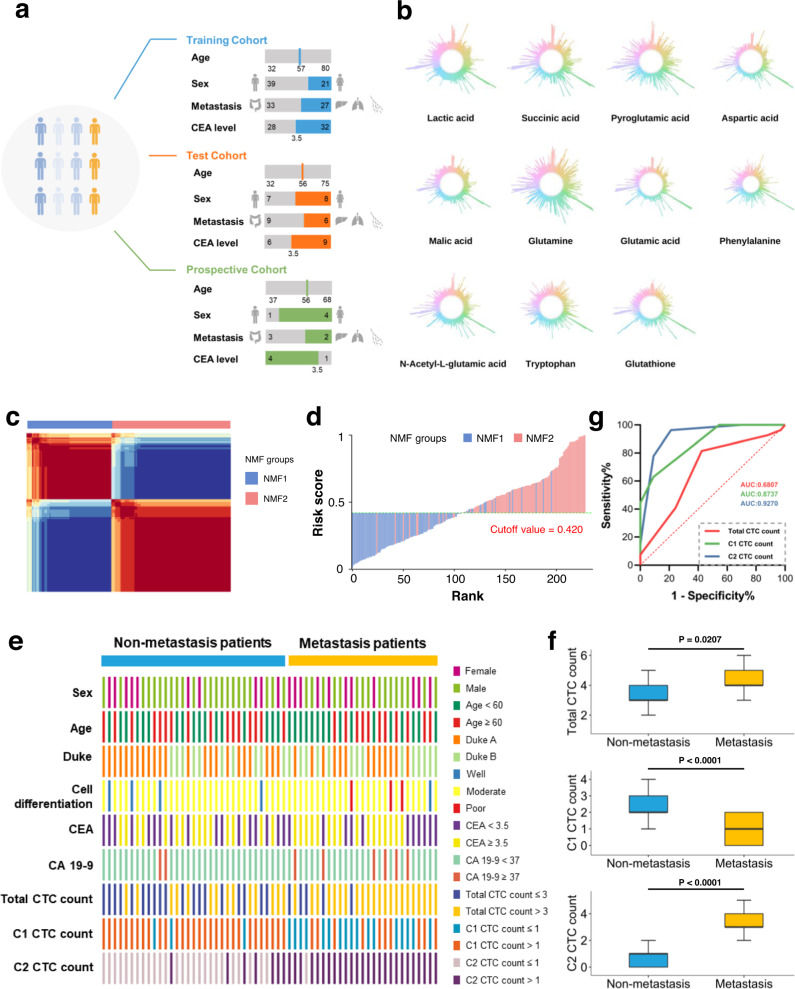
Discovery of CTC metabolic fingerprints based on abundance of target metabolites in single CTCs to predict colorectal cancer metastasis potential. **a** Clinical characteristics and biomarker levels of enrolled CTC-positive colorectal cancer patients. **b** Representative abundance of 11 target metabolites in single CTCs. **c** NMF clustering of single CTCs. Based on the obtained cophenetic correlation coefficients shown in Supplementary Fig. 11, *K* = 2 was considered as the preferred cluster and **d** risk score plot of single CTCs. The risk score was constructed as follows: Log_10_(risk score/(1-risk score)) = −(0.932 × abundance of glutamic acid) + (3.967 × abundance of malic acid) − (0.166 × abundance of aspartic acid) − (1.822 × abundance of lactic acid) − 3.694. Obviously, all CTCs could be split into two groups: low risk (risk score <0.420) and high risk (risk score ≥ 0.420) based on the cutoff value using the Youden index. **e** Correlation of patient metastasis status (metastasis vs. non-metastasis) with clinical characteristics and biomarker levels, and the number of CTCs in cell subgroups (C1 CTC count, C2 CTC count, and total CTC count) in the training cohort. **f** Comparison of C1 CTC count, C2 CTC count and total CTC count between non-metastasis and metastasis groups in the training cohort. The black line represents the median. The top and bottom of the box represent the 75th and 25th quartiles while the whiskers represent 1.5× interquartile range. Patients with non-metastasis, *n* = 33; patients with metastasis, *n* = 27. The two-tailed Student’s *t* test was used to determine statistical significance. Total CTC count, metastasis group vs. non-metastasis group, *p* = 0.0207; C1 CTC count, metastasis group vs. non-metastasis group, *p* < 0.0001; C2 CTC count, metastasis group vs. non-metastasis group, *p* < 0.0001. **g** ROC analysis of C1 CTC count, C2 CTC count and total CTC count in predicting metastasis in the training cohort. Some graphical elements in (**a**) were created with BioRender.com (accessed on 24 March 2023). NMF non-negative matrix factorization, AUC area under curve, CEA carcinoembryonic antigen, CA 19-9 carbohydrate antigen 19-9.

In our study, 208 colorectal cancer patients are enrolled. After CTC enrichment and identification (Supplementary Figs. 7 and 8), 83 patients were CTC-positive and 125 patients were CTC-negative, among which 75 CTC-positive patients and 115 CTC-negative patients were followed up for 2 years. In these 75 patients, we randomly selected 80% of patients (60) as the training cohort and the remaining 20% of patients (15) as the test cohort (Fig. 4a). The primary clinical characteristics and biomarker levels of the patients are shown in Supplementary Table 4. In the training cohort, the detected number of CTCs ranged from one to six per 15 ml of blood, distributed as 1 (3.3%), 2 (6.7%), 3 (30.0%), and ≥4 (60.0%). There was a significant difference in the total CTC count between the non-metastasis group (3 (3–4)) and metastasis group (4 (4–5)), represented by the median value (the first quartile IQ1- the third quartile IQ3) (*p* = 0.0207). In the test cohort, 1 (0.0%), 2 (20.0%), 3 (46.7%) and ≥4 (33.3%) were detected. A significant difference between the non-metastasis group and metastasis group was also observed, with a *p* of 0.0473.

The levels of 11 target metabolites were determined in each CTC using single-cell quantitative mass spectrometry developed above. The abundance of 11 metabolites in single CTCs was observed and is presented in Fig. 4b. In Supplementary Fig. 9, we also depicted the concentration distribution of each metabolite. Taking glutathione as an example, its concentration in individual CTCs ranged from 0.22 to 4.33 mM, with intrapatient RSD up to 124.3%, again confirming a more significant heterogeneity within CTCs from the same patient. Notably, the time from blood collection to picking and analysis of cells (~2 h) did not affect the stability of the target metabolites significantly in this study (Supplementary Fig. 10).

To preserve the heterogeneous characteristics of CTCs during modeling, we applied an unsupervised learning algorithm—non-negative matrix factorization (NMF) coupled with logistic regression to divide total CTCs into groups based on the quantitative level of target metabolites in the training cohort. After calculating cophenetic correlation coefficients (Supplementary Fig. 11), the optimal number of cell subgroups was 2 (Fig. 4c). Four metabolites (glutamic acid, lactic acid, aspartic acid and malic acid) were identified as important factors, and the risk score of each CTC was calculated. An optimal cutoff value of 0.420 was selected using the Youden index (Fig. 4d). Using the 4-metabolite fingerprint classifier, the CTCs were divided into two subgroups, C1 (<cutoff) and C2 (≥cutoff). The abundance of 4 metabolites in C1 and C2 subgroups is shown in Supplementary Fig. 12. Total CTC count and C1 CTC count, C2 CTC count of each patient in the training cohort are summarized in Supplementary Table 5. Meanwhile, an support vector machine (SVM)-based machine learning method^29^ was tested, and the detailed information is shown in Supplementary Tables 6 and 7. The unsatisfactory performance of this method may result from its hypothesis assuming the homogeneity of CTCs in each patient.

### Correlation of CTC subgroups with colorectal cancer metastatic risk

Taking the incidence of metastasis during the follow-up into account, no significant association was observed between colorectal cancer metastatic risk and the clinical characteristics and common cancer biomarker levels of the patients, such as age, sex, Duke, grade of differentiation, carcinoembryonic antigen (CEA), carbohydrate antigen 19-9 (CA 19-9) in the training cohort (Fig. 4e). However, higher total CTC count (*p* = 0.0207), lower C1 CTC count (*p* < 0.0001), and higher C2 CTC count (*p* < 0.0001) were associated with metastatic risk (Fig. 4f). According to the Youden index using ROC curves (Fig. 4g), the optimal cutoff values were 3 for total CTC count, 1 for C1 CTC count and 1 for C2 CTC count. The patients with a total CTC count/C2 CTC count higher than the cutoff value and a C1 CTC count lower than the cutoff value were considered unfavorable (i.e., had a greater tendency to metastasize) (Fig. 4e). Among these three classifiers, a total CTC count of 3 yielded a sensitivity, specificity and accuracy of 57.6%, 81.5% and 68.3%, respectively. The area under the ROC curve (AUC) was only 0.681, clearly pointing out the presence of false-positives and false negatives in predicting the incidence of metastasis with total CTC count. Comparatively, a C2 CTC count of 1 yielded a sensitivity, specificity, accuracy and AUC with the highest quality of 78.8%, 96.3%, 86.7% and 0.927, respectively, suggesting its predictive ability. This finding was also confirmed by univariate and multivariate logistic regression analyses involving CTC counts, clinical characteristics and biomarker levels (Supplementary Table 8).

**Fig. 5:**
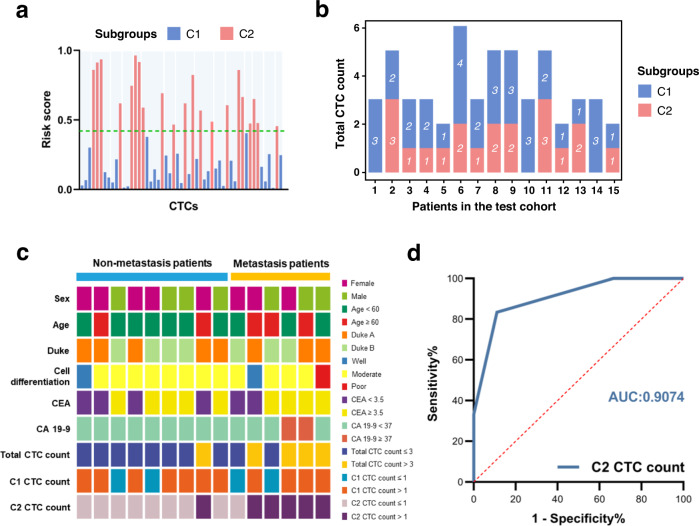
Validation of the performance of CTC metabolic fingerprints. **a** Risk score of single CTCs in the test cohort using the 4-metabolite classifier. Two subgroups were also identified. **b** The distribution of C1 CTC count and C2 CTC count in individual patients. **c** Correlation of patient metastasis status (metastasis vs. non-metastasis) with clinical characteristics and biomarker levels, and the number of CTCs in cell subgroups (C1 CTC count, C2 CTC count, and total CTC count) in the test cohort. **d** ROC curve of C2 CTC count in predicting metastasis in the test cohort. AUC area under curve.

To further validate the performance of the 4-metabolite fingerprint classifier, we applied it to the test cohort. The results indicated that 53 CTCs could also be separated into two subgroups (Fig. 5a), and the distributions of C1 CTC count and C2 CTC count in individual colorectal cancer patients are clearly shown in Fig. 5b. Consistent with the training cohort, there was no significant association of metastatic risk with the clinical characteristics and biomarker levels, total CTC count and C1 CTC count of the patients. The C2 CTC count played a significant role (Fig. 5c) and achieved good sensitivity, specificity, accuracy and AUC (Fig. 5d). It is deserved to mention that 57.9% (33/57) of metastatic patients were accurately predicted in all the enrolled patients using our molecular typing system. The false-positive and false-negative rates of metastasis were 19.0 and 6.1% in CTC-positive patients. Interestingly, 20.9% (24/115) patients in CTC-negative group also developed metastasis. Because our molecular typing system was not used for these patients, the inflated false-negative rate was probably due to the limitation of the currently available CTC enrichment method^30,31^ and thus the overcounting in CTC-negative patients.

Finally, the association of the discovered metabolic phenotype with the metastatic potential of cells was validated from a functional aspect. Treatment of cells with four metabolites significantly promoted cell metastasis, as shown by transwell assay^32^ (Supplementary Fig. 13). Moreover, we established two cell lines from CTCs in C1 and C2 subgroups individually. In agreement with previous reports^33^, long term CTC culture (i.e., >6 months) could be established (Supplementary Fig. 14a). Using these derived cells, we further confirmed that C2 subgroup had a greater proliferative capacity using CCK-8 assay (Supplementary Fig. 14b) and a higher migratory capacity using transwell assay (Supplementary Fig. 14c). Finally, we constructed a CTC-derived explant (CDX)^33,34^. As a result, the mice in C2 subgroup showed a more aggressive tumor phenotype as compared to those in C1 subgroup, with more metastatic foci in lung and liver (Supplementary Fig. 14d, e). In addition, histological dissection was carried out and the result showed the incidence of metastatic tumors (Supplementary Fig. 14f). These in vivo findings provided further evidence for the association of the metabolic phenotype with the metastatic potential of CTC subgroups. Therefore, the C2 CTC count classified by the 4-metabolite fingerprint may be a potential tool for predicting the incidence of colorectal cancer metastasis.

### Prospective clinical study using CTC subgroups classified by metabolic fingerprinting

In this study, we finally performed a prospective study of 15 colorectal cancer patients with no metastasis. Among these patients, five patients (33.3%) were CTC-positive before the operation, with a median CTC count of 4/15 ml of blood (range 3–5). The basic clinical information of these five patients is summarized in Supplementary Table 9.

**Fig. 6:**
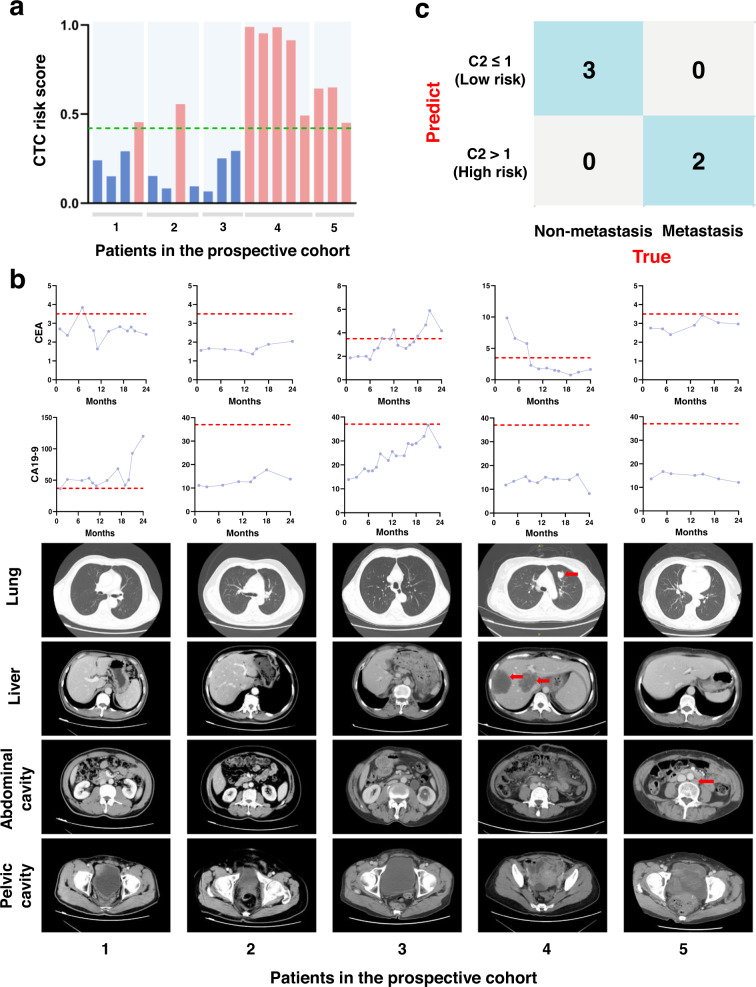
Prospective clinical study of CTC metabolic fingerprints. **a** Risk score of single CTCs in the prospective cohort using the 4-metabolite classifier. **b** Abundance of common cancer biomarkers (CEA and CA 19-9) during the follow-up of five patients in the upper panels. The cutoff values of CEA and CA 19-9 are indicated by a red line. The lower panels show the corresponding CT images. Metastasis is visible and indicated by red arrows (patient 4 and patient 5). **c** The confusion matrix of the patients using C2 CTC count. CEA carcinoembryonic antigen, CA 19-9 carbohydrate antigen 19-9.

After target metabolite quantification at the single-CTC level and extraction of C2 subgroup in each patient, we found that patients 1–3 had a C2 CTC count of 1, while patients 4–5 had a C2 CTC count higher than the cutoff value and were considered to have the potential for metastasis (Fig. 6a). During the 2-year follow-up period, patient 4 experienced liver and lung metastasis at month 15, and patient 5 developed lymph node metastasis at month 8, whereas the other 3 patients had no evidence of imaging characteristics of any metastasis. In addition, no significant correlation was found between cancer biomarkers, including CEA and CA 19-9, and metastatic risk (Fig. 6b). This result further validated the reliability of the C2 subgroup classified by the 4-metabolite fingerprint to predict the incidence of metastasis, rather than other currently available indices (Fig. 6c).

## Discussion

In this study, we initially found 2547 and 3606 significant metabolic features in the two pairs of cells associated with colorectal cancer metastasis using mass spectrometry-based untargeted metabolomic analysis, then identified 14 shared metabolites significantly enriched in metabolic pathway enrichment analyses, further quantified 11 target metabolites in single CTCs by setting up a home-built single-cell quantitative mass spectrometry platform, and finally built a 4-metabolite fingerprint classifier of CTCs for predicting colorectal cancer metastatic risk within 2 years. Using the 4-metabolite fingerprint classifier, CTCs can be divided into two subgroups, C1 and C2, which were functionally evaluated by several in vitro and in vivo assays. Patients with more than 1 CTC in the C2 subgroup had a higher incidence of metastasis. After development in the training cohort and validation in the test cohort and the prospective cohort, the developed molecular typing system showed a better predictive ability of metastatic risk than other indices, including total CTC count. On the other hand, cell grouping results evidently highlighted CTC heterogeneity even within the same patient. This CTC heterogeneity can be well discerned with our home-built single-cell quantitative mass spectrometry platform.

To date, cultured cell lines are still good models for metabolomics studies, given the complexity of establishment, characterization and culture of patient-derived CTCs^35^. In the untargeted metabolomic profiling of colorectal cancer cell lines with differential metastatic potential, the identified metabolites were concentrated in interactive metabolic pathways, exhibiting statistically significant alterations that correlated with the metastatic phenotype. Accordingly, we assembled a metabolic map depicting these changes in three main pathways: amino acid metabolism, glutathione metabolism, and the Warburg effect. Based on this knowledge-based dimensionality reduction, redundant data can be excluded and data interpretation can be simplified in a biologically meaningful context^36^. Specifically, the enriched pathways containing the selected metabolites are fit to some extent what is known of key adaptive metastatic pathways in this way. As it is well known, glutathione is composed of glutamic acid and the other two amino acids by peptide bonds condensation. Glutathione metabolism is closely related with cancer metastasis and metastatic cancer treatment^37^. In addition, amino acid metabolism involving aspartic acid and glutamic acid can also promote cancer metastasis^38^. Moreover, the Warburg effect contributing to enhanced malic acid and lactic acid, fuels cancer metastasis and can mitigate oxidative stress to help cancer cells to survive the stringent metastatic process^39^. To investigate the selected metabolites in these pathways to predict the metastatic potential of CTCs, they were then subjected to the home-built single-cell quantitative mass spectrometry platform.

In general, in cell population analysis, single-cell information is easily overwhelmed in pooled cells^40^. For CTCs, data from this study and previous studies both suggested the presence of significant heterogeneity in the cell population. Thus, analysis of CTCs at the single-cell level is more desired and attractive. However, translation from the macroscopic level to the microscopic level remains a practical and technical challenge as mentioned earlier. These challenges were well addressed on our home-built single-cell quantitative mass spectrometry platform. First, a single cell is typically micrometer-sized and has a volume in the picoliter range. It is normally difficult to extract cellular contents from a single cell with minimal perturbation. To date, several methods have been developed to collect single-cell material^41,42^, for example, micro-sampling technique coupled with mass spectrometry in single-cell metabolomic studies^43–45^, single-cell analysis of drug distribution^46–48^, and etc. In this study, electro-osmotic extraction using a finely pulled nanocapillary tip enabled the withdrawal of a subpicoliter volume of cellular contents. The extracted volume can be controlled by varying the applied voltage and time between the working electrode and the reference electrode. Second, untargeted metabolomic profiling at the single-cell level has been previously reported^49^. However, these studies often focused on chemical characterization, provided semiquantitative information, and suffered from unsatisfying repeatability^50^. In comparison, mass spectrometry-based targeted analysis could be a quantitative tool in single-cell analysis because of its high sensitivity, good reproducibility and remarkable quantification capability^51^, which may be more compatible with clinical practice for marker development and therapeutic target validation^20^. Third, the only major concern of the single-cell quantitative mass spectrometry platform is that it can be sensitive to nonbiological variations, including both technical variability arising through technical effects and confounding factors among which batch effect experienced between biological replicates is perhaps the most obvious one^26^. After careful optimization of the platform, nonbiological variations were well reduced and good repeatability and sensitivity were achieved.

During the metabolic data analysis of single CTCs or even in the whole work, inclusion of cell heterogeneity in data mining may be the most challenging part. Theoretically, there are two aspects that should be considered: (1) direct use of machine learning methods based on cell homogeneity may be problematic; (2) an unsupervised CTC clustering method may be needed to distinguish the contribution of specific CTC characteristics to metastatic potential. In this work, the methods using either total CTC count or metabolic profile showed poor predictive performance, as long as cell homogeneity was assumed. To incorporate cell heterogeneity into our data, we employed an unsupervised clustering method, NMF, which is an efficient machine learning approach for cell grouping^52^. Indeed, this clustering method has been applied in previous studies using heterogeneous data^53^. Finally, two subgroups of CTCs were found by NMF, primarily with four metabolites. Among these 4 metabolites, glutamic acid is in glutathione metabolism pathway, glutamic acid and aspartic acid are closely related to amino acid metabolism, and glutamic acid, malic acid and lactic acid are in the Warburg effect^54^.

Using this 4-metabolite classifier, the number of CTCs in the C2 subgroup was significantly associated with metastatic risk. Positive results were also obtained in the test and prospective cohorts. The prediction performance of our molecular typing system was better than that of total CTC count or metabolite profile only and better than that of clinical indices. Indeed, the four metabolites have been previously reported to participate in cancer metastasis. For example, glutamic acid is a major bioenergetic substrate for proliferating cancer cells and actively modulates cancer cell metastasis through regulating metabolic pathways^55^. Previous reports have also shown that glutamic acid level was markedly elevated in metastatic cancer patients^56^. Aspartic acid is also closely relevant to metastatic events occurring in vivo^57^ and intracellular synthesis of aspartic acid played a large role in metastasis of colorectal cancer cells^58^. Lactic acid is a product of glycolysis (also known as the Warburg effect under aerobic condition) capable of promoting oncogenic progression and cancer metastasis^59^. Furthermore, malic acid can contribute to the Warburg effect and was found to be significantly enhanced in cancer metastasis^60^. More importantly, increasing evidence has revealed an activation of the Warburg effect^16,17^ and glutathione metabolism in CTCs^17,61^. Consistent with previous observations, the findings of in vitro and in vivo experiments on cultured cells, CTC-derived cells and CDX model in this study provided evidence that metabolic phenotype had an influence on the metastatic potential of cells and also supported the biological function of four metabolites in cell metastasis to some extent.

However, the underlying metabolic mechanism is still far-reaching. Follow-up experiments are needed to assign more biological meaning to the metabolites and to move toward finding mechanisms of colorectal cancer metastasis before clinical translation and utility, which could include using pathway metabolites, pathway inhibitors and stable isotope labeling as well as flux analysis to reveal biological function of specific metabolites^62^, and performing biopsy of metastatic lesions in the context of clinical trials to reveal the association of metabolic profiles between the tumor at secondary sites and CTCs in CTC-positive patients, and the tumors at secondary sites between CTC-positive and CTC-negative patients.

In conclusion, molecular typing at the single-CTC metabolite level is a creative step toward a more precise prediction of metastasis potential in colorectal cancer patients. The realization of this step was enabled by the successful setup of the home-built single-cell quantitative mass spectrometry platform and the inclusion of cell heterogeneity in the prediction model. Although full validation of this CTC metabolic fingerprint classifier and subsequent CTC subgrouping is required to elucidate the clinical utility of this molecular typing system, and the studies with a higher statistical power such as randomization studies are required to evaluate this causal relationship, this is an interesting report that single CTC heterogeneity at the metabolite level was involved in clinical management. With the maturation of this approach, molecular typing at the single-cell level may become more competitive in specific clinical situations and we believe that the clinical relevance of any molecular-typed CTC subgroup with disease progression and patient outcome will ultimately benefit the real-life clinical practice. Furthermore, metabolic grouping approach based on single-cell information may help provide more insight into cancer research and offer more potential clinical markers and therapeutic targets. It may also broaden the window of opportunity for modifying clinical intervention to slow or prevent cancer progression.

## Methods

### Ethical statement

This study was approved by the Institutional Review Board of Jiangsu Cancer Hospital and Sir Run Run Hospital Affiliated to Nanjing Medical University, Nanjing, China. All the animal experiments were approved by Institutional Animal Care and Use Committee of NMU and performed according to the ARRIVE guidelines.

### Cell sample preparation and mass spectrometry-based untargeted metabolomic analysis

Two hours before metabolite extraction, the cell culture medium was replaced with fresh medium. After discarding the medium in each culture dish, the cells were quickly rinsed twice with cold isotonic saline (0.9% NaCl (w/v), 4 °C). Water (1.5 ml) was added to each dish, and then the dishes were stored in a freezer (−80 °C) for 20 min before extraction. The cells were completely collected with a cell scraper. A fraction of the cell lysate (20 μl) was subjected to bicinchoninic acid (BCA) protein assay, and the remaining cell lysate was extracted by the addition of 4.5 ml of methanol containing 3 μg of internal standard (acetaminophen). Finally, the cell lysate in each dish was transferred to an Eppendorf tube, vigorously vortexed for 5 min, and then centrifuged at 10,000 × *g* for 10 min at 4 °C. The supernatant was transferred to another tube and evaporated to dryness in a refrigerated CentriVap benchtop vacuum concentrator (Labconco Corporation, Kansas, USA). The residue was reconstituted with methanol-water (75:25) and centrifuged twice at 12,000 × *g* for 15 min at 4 °C.

The metabolomic analysis was performed by ultra-performance liquid chromatography-tandem high-resolution mass spectrometry (UPLC-HRMS). Chromatographic separation of the metabolites was carried out on a UPLC Ultimate 3000 system (Dionex, Germering, Germany) equipped with a Phenomenex Kinetex C18 column (2.1 mm × 100 mm, 2.6 μm, 100 Å). Water (A) and acetonitrile (B), both containing 0.1% formic acid (v/v), were used as the mobile phases. Under a flow rate of 0.4 ml/min, the cellular metabolites were eluted with a gradient program as follows: 10% B (0 min) → 30% B (1 min) → 95% B (19 min) → 95% B (20 min). The column oven temperature was maintained at 40 °C, and all samples were maintained at 4 °C during the whole analysis. All samples were analyzed in a randomized fashion to avoid complications of the injection order. Pooled samples were used as quality control sample (QC) covering the whole analytical process. QC sample was injected every 5th cellular sample followed by a blank (i.e., sample preparation protocol applied to a water sample).

A Q-Exactive mass spectrometer (Thermo Fisher Scientific, Bremen, Germany) equipped with an electron spray ionization (ESI) source was used for MS detection, and both positive and negative ion modes were employed. MS parameters were set as follows: spray voltage, +3.5 kV for positive and −2.5 kV for negative; capillary temperature, 300 °C; aux gas flow rate (arb), 13. Vacuum was typically below 5 × 10^−10^ mbar. A full mass scan (*m*/*z* 67–1000) used a resolution of 70,000 with an automatic gain control (AGC) target of 1 × 10^6^ ions and a maximum ion injection time of 100 ms. Data-dependent MS/MS was acquired in the “Top10” data-dependent mode. For the product ion scan type, the resolution was set at 17,500, the automatic gain control target was 1 × 10^5^ ions, and the maximum ion injection time was 50 ms. The eligible precursor ions were fragmented between 15 and 45 normalized collision energies (NCEs) in increments of 15. All operations and acquisitions were controlled by Xcalibur 2.0.7 (Thermo Fisher Scientific, Bremen, Germany).

### Metabolomic data processing

The raw data obtained from UPLC-HRMS were processed using MS-DIAL v4.80 software^63^. Pretreatment procedures were performed, including peak finding, alignment, filtering and normalization. More specifically, the peaks that exceeded 10,000 in height were automatically extracted and aligned across the samples. The peaks detected in less than 80% of samples in all groups^64^ or that had a maximum intensity of less than 3-fold S/N were removed. The noise threshold was estimated by using the average signal across all blank runs. The ion intensities were normalized by protein amount, internal standard acetaminophen (*m*/*z* 150.0548 for negative ion mode, *m*/*z* 152.0701 for positive ion mode) and pooled sample as quality control^65^. The resulting data could be converted to a matrix, which mainly included the sample name, *m*/*z* and adduct type, and normalized ion intensity. We also imputed the missing values with the 1/10 minimum area value of peaks.

We applied PCA with SIMCA 14.1 software (Umetrics, Umea, Sweden) to evaluate the overall distribution of data and check the run quality. Further OPLS-DA with SIMCA 14.1 software was utilized to identify the metabolites that were significantly different in two pairs of colorectal cancer cell lines (SW480 vs. SW620 and HT-29 vs. COLO 205). The Log_2_ fold change (Log_2_FC) and *p* were subsequently determined on the acquired metabolomic data, and the metabolic features with |Log_2_FC|> 1.0, *p* < 0.05, and variable importance in projection (VIP) value >1 were considered significant. Molecular identification of the significantly changed metabolites was achieved by comparing the MS/MS fragmentation spectra of metabolic features against MS-DIAL internal database or matching the retention time and MS accurate mass of features to metabolite standards. The intersection of the identified metabolites with significantly differential abundance obtained from the two pairs of cell lines was selected. We then utilized MetaboAnalyst (http://www.metaboanalyst.ca/) to perform KEGG pathway analysis and MSEA on the selected metabolites^66^. The corresponding figures were visualized by the R programming language (R version 4.0.3).

### Capillary preparation for single-cell manipulation

Single-cell manipulation was performed using a pair of nanocapillary tips and microcapillary holders. The nanocapillaries were pulled from borosilicate glass capillaries (i.d. = 0.58 mm, o.d. = 1 mm, with filament) with a Sutter P-2000 puller (Sutter Instrument, Novato, USA) to make the nanospray tips suitable for MS ionization. A two-step pulling program was set as follows: HEAT = 350, FIL = 3, VEL = 30, DEL = 220, PULL = NA; HEAT = 350, FIL = 3, VEL = 40, DEL = 180, PULL = 120. The prepared nanocapillaries were characterized by SEM (JSM 7800F, JEOL Ltd., Tokyo, Japan). The microcapillaries were pulled from borosilicate glass capillaries (i.d. = 0.58 mm, o.d. = 1 mm, with filament) with a Sutter P-1000 puller (Sutter Instrument, Novato, USA) to make the microcapillary holders. The single-step pulling program was as follows: HEAT = 520, PUL = 100, VEL = 30, TIME = 250, PRESSURE = 500. The pulled capillaries were further processed with a Microforge (MF2, Narishige, Tokyo, Japan) to make the holders ∼1 μm open.

### Single-cell micro-sampling and single-cell quantitative mass spectrometry

The microcapillary holder was connected to a microinjector (IM-12, Narishige, Tokyo, Japan) to fix the suspended CTCs, and the nanocapillary tip was combined with an insulated fixator for cellular extraction. Both the nanocapillary tip and microcapillary holder were operated with a motorized micromanipulator system (NTX-N4, Nikon, Tokyo, Japan) mounted on an inverted microscope (Ti2-U, Nikon, Tokyo, Japan). An Ag/AgCl wire as the working electrode was inserted in the nanocapillary tip and connected to an electrochemical station (CHI 660, CH Instruments, Austin, USA). Another Ag/AgCl wire as the reference electrode was used to form an electrical loop to introduce electro-osmosis assisted cellular extraction (Supplementary Fig. 2). Cellular extraction was performed followed by sequential extraction of ~120 fL of 1 mM isotope-labeled internal standard solution. After sampling, the nanocapillary tip was relocated in front of the mass spectrometer inlet with a distance of ~5 mm. An AB SCIEX QTRAP 5500 MS/MS system was used in this study for target molecule quantification. Multiple reaction monitoring (MRM) transitions were optimized for each analyte. The instrument parameters of mass spectrometry were as follows: curtain gas (CUR) = 10, ion spray voltage (IS) = −2250, TEM = 0, ion source gas 1 (GS1) = 0 and ion source gas 2 (GS2) = 0.

### Reporting summary

Further information on research design is available in the Nature Portfolio Reporting Summary linked to this article.

## Supplementary information

Supplementary Information

Reporting Summary

## Data Availability

The summary statistics for the metabolites that displayed significant differences in cells are shown in Supplementary Table 1 and Source Data. The datasets are available in the Metabolomics Workbench database under the accession number ST002341. Source data are provided with this paper.

## Source data

Source Data
