# Supplementary material for: Single-cell metabolic fingerprints discover a cluster of circulating tumor cells with distinct metastatic potential
Source: Nat Commun. 2023 Apr 29;14:2485. doi: 10.1038/s41467-023-38009-3 (PMC10148826; doi:10.1038/s41467-023-38009-3)
Supplement: Supplementary file 1 — Supplementary Information [file 41467_2023_38009_MOESM1_ESM.pdf]

Supplementary Information

Single-Cell Metabolic Fingerprints Discover a Cluster of Circulating Tumor Cells with Distinct Metastatic Potential

Wenjun Zhang[1,7], Feifei Xu[1,7], Jiang Yao[1], Changfei Mao[2], Mingchen Zhu[3], Moting Qian[1], Jun Hu[1], Huilin Zhong[4], Junsheng Zhou[4], Xiaoyu Shi[1], Yun Chen[1,5,6]

[1] School of Pharmacy, Nanjing Medical University, Nanjing, 211166, China

[2] Department of General Surgery, Jiangsu Cancer Hospital (Jiangsu Institute of Cancer Research, Nanjing Medical University Affiliated Cancer Hospital), Nanjing, 210009, China

[3] Department of Clinical Laboratory, Jiangsu Cancer Hospital (Jiangsu Institute of Cancer Research, Nanjing Medical University Affiliated Cancer Hospital), Nanjing, 210009, China

[4] School of Computer Science and Technology, Nanjing Normal University, Nanjing, 210046, China

[5] State Key Laboratory of Reproductive Medicine, Nanjing, 211166, China

[6] Key Laboratory of Cardiovascular and Cerebrovascular Medicine, Nanjing, 211166, China

[7] These authors contributed equally: Wenjun Zhang, Feifei Xu.

Correspondence e-mail: ychen@njmu.edu.cn

Supplementary Table 1. Details of the metabolites with significantly differential abundance in two pairs of cell lines. **a** SW480 vs. SW620 and **b** HT-29 vs. COLO 205. Statistical analysis was performed using the two-tailed Student's t-test. VIP variable important in projection, FC fold change.

a

Metabolite	m/z (measured)	m/z (calculated)	ppm	Formula	Log_2 FC	P	VIP
Alanine	88.0395	88.0393	1.65	$[C_3H_7NO_2-H]^-$	2.45	2.64E-03	1.84
Lactic acid	89.0234	89.0233	0.67	$[C_3H_6O_3-H]^-$	1.65	2.52E-02	1.46
Phosphoric acid	96.9685	96.9685	-0.53	$[H_3O_4P-H]^-$	1.64	1.21E-02	1.54
Uracil	111.0187	111.0189	-2.02	$[C_4H_4N_2O_2-H]^-$	1.62	1.07E-02	1.55
Fumaric acid	115.0025	115.0026	-0.91	$[C_4H_4O_4-H]^-$	1.31	2.36E-02	1.35
Proline	116.0710	116.0706	3.32	$[C_5H_9NO_2+H]^+$	3.00	3.80E-03	1.12
Succinic acid	117.0181	117.0182	-1.58	$[C_4H_6O_4-H]^-$	1.87	2.48E-02	1.58
Cysteine	122.0271	122.0270	0.53	$[C_3H_7NO_2S+H]^+$	1.48	3.16E-02	1.23
Taurine	124.0061	124.0063	-1.78	$[C_2H_7NO_3S-H]^-$	1.27	1.00E-02	1.25
Pyroglutamic acid	128.0341	128.0342	-1.01	$[C_5H_7NO_3-H]^-$	1.56	2.60E-03	1.53
Aspartic acid	132.0291	132.0291	0.04	$[C_4H_7NO_4-H]^-$	3.02	6.71E-03	2.03
Malic acid	133.0132	133.0131	0.60	$[C_4H_6O_5-H]^-$	2.03	3.91E-02	1.64
Glutamine	145.0609	145.0608	0.77	$[C_5H_{10}N_2O-H]^-$	2.64	2.61E-02	1.74
Glutamic acid	146.0449	146.0448	0.52	$[C_5H_9NO_4-H]^-$	2.84	1.49E-02	1.85
Methionine	150.0587	150.0583	2.29	$[C_5H_{11}NO_2S+H]^+$	2.18	4.73E-03	1.10
Xanthine	151.0250	151.0251	-0.41	$[C_5H_4N_4O_2-H]^-$	1.56	2.13E-03	1.46
Histidine	154.0613	154.0611	1.02	$[C_6H_9N_3O_2-H]^-$	1.92	2.14E-03	1.69
Orotic acid	155.0087	155.0087	-0.54	$[C_5H_4N_2O_4-H]^-$	3.00	6.76E-03	1.77
Phenylalanine	164.0707	164.0706	0.27	$[C_9H_{11}NO_2-H]^-$	2.78	1.55E-02	1.77
3-Methylxanthine	165.0407	165.0407	-0.13	$[C_6H_6N_4O_2-H]^-$	1.19	1.03E-02	1.47
Pyridoxine	170.0810	170.0812	-0.76	$[C_8H_{11}NO_3+H]^+$	2.91	2.52E-02	1.17
N-Acetylleucine	172.0972	172.0968	2.09	$[C_8H_{15}NO_3-H]^-$	2.00	1.93E-02	1.75
Glucose	179.0550	179.0550	0.03	$[C_6H_{12}O_6-H]^-$	1.81	1.44E-02	1.55
N-Acetyl-L-glutamic acid	188.0554	188.0553	0.06	$[C_7H_{11}NO_5-H]^-$	3.45	9.13E-03	2.15
N-Acetyl-L-methionine	192.0692	192.0689	1.61	$[C_7H_{13}NO_3S+H]^+$	2.49	1.38E-02	1.70
N-Acetylhistidine	198.0877	198.0873	1.98	$[C_8H_{11}N_3O_3+H]^+$	-1.65	2.59E-02	1.12
Tryptophan	203.0820	203.0815	2.44	$[C_{11}H_{12}N_2O_2-H]^-$	1.94	6.59E-03	1.58
N-Acetyl-L-phenylalanine	206.0819	206.0812	3.74	$[C_{11}H_{13}NO_3-H]^-$	1.75	1.75E-02	1.51
Pantothenic acid	218.1030	218.1023	3.40	$[C_9H_{17}NO_5-H]^-$	2.42	2.97E-03	1.86
Leucylproline	229.1549	229.1547	0.96	$[C_{11}H_{20}N_2O_3+H]^+$	2.93	1.99E-02	1.14
Uridine	243.0613	243.0612	0.69	$[C_9H_{12}N_2O_6-H]^-$	1.95	1.58E-03	1.63
Leucylleucine	245.1858	245.1860	-0.89	$[C_{12}H_{24}N_2O_3+H]^+$	4.38	6.46E-03	1.55
Adenosine	268.1041	268.1040	0.11	$[C_{10}H_{13}N_5O_4+H]^+$	9.00	2.42E-02	1.81
Guanosine	282.0839	282.0833	2.00	$[C_{10}H_{13}N_5O_5-H]^-$	1.48	1.52E-02	1.21
	284.0985	284.0989	-1.64	$[C_{10}H_{13}N_5O_5+H]^+$	2.84	6.25E-03	1.28
Xanthosine	283.0682	283.0673	2.97	$[C_{10}H_{12}N_4O_6-H]^-$	1.81	1.25E-02	1.19
Glutathione	306.0756	306.0754	0.51	$[C_{10}H_{17}N_3O_6S-H]^-$	3.90	2.26E-04	2.10
Uridine 5'-monophosphate	323.0283	323.0275	2.47	$[C_9H_{13}N_2O_9P-H]^-$	2.09	8.28E-03	1.50
Riboflavin	375.1296	375.1299	-0.72	$[C_{17}H_{20}N_4O_6-H]^-$	2.38	2.96E-02	1.10
	377.1455	377.1456	-0.06	$[C_{17}H_{20}N_4O_6+H]^+$	2.41	5.50E-03	1.37
	399.1282	399.1275	1.82	$[C_{17}H_{20}N_4O_6+Na]^+$	1.71	5.21E-03	1.16
Deoxycholic acid	391.2849	391.2843	1.67	$[C_{24}H_{40}O_4-H]^-$	-2.87	3.01E-02	1.73
LPC 18:1	522.3570	522.3554	3.01	$[C_{26}H_{52}NO_7P+H]^+$	1.56	3.01E-02	1.13
Uridine diphosphate glucose	565.0472	565.0466	0.94	$[C_{15}H_{24}N_2O_{17}P_2-H]^-$	3.51	1.30E-02	2.33
Oxidized glutathione	611.1447	611.1436	1.84	$[C_{20}H_{32}N_6O_{12}S_2-H]^-$	1.49	4.66E-02	1.10

b

Metabolite	m/z (measured)	m/z (calculated)	ppm	Formula	Log_2 FC	P	VIP
Alanine	88.0395	88.0393	1.65	$[C_3H_7NO_2-H]^-$	1.93	2.25E-05	1.22
Lactic acid	89.0234	89.0233	0.67	$[C_3H_6O_3-H]^-$	3.40	7.87E-05	1.64
Serine	104.0341	104.0342	-0.96	$[C_3H_7NO_3-H]^-$	3.26	4.66E-04	1.08
Histamine	110.0711	110.0713	-1.49	$[C_5H_9N_3-H]^-$	7.24	1.46E-05	1.70
Succinic acid	117.0181	117.0182	-1.58	$[C_4H_6O_4-H]^-$	1.26	1.55E-02	1.08
Threonine	118.0498	118.0499	-0.50	$[C_4H_9NO_3-H]^-$	2.86	4.86E-04	1.01
Pyroglutamic acid	128.0341	128.0342	-1.01	$[C_5H_7NO_3-H]^-$	1.35	3.58E-06	1.27
Ornithine	131.0816	131.0815	0.43	$[C_5H_{12}N_2O_2-H]^-$	-4.52	3.99E-08	1.38
Aspartic acid	132.0291	132.0291	0.04	$[C_4H_7NO_4-H]^-$	1.08	3.63E-04	1.06
Creatine	132.0768	132.0768	0.20	$[C_4H_9N_3O_2+H]^+$	-3.10	1.00E-08	1.51
Malic acid	133.0132	133.0131	0.60	$[C_4H_6O_5-H]^-$	1.71	6.81E-04	1.15
Adenine	134.0461	134.0461	0.14	$[C_5H_5N_5-H]^-$	4.65	1.81E-06	1.38
α-Ketoglutaric acid	145.0131	145.0131	-0.62	$[C_5H_6O_5-H]^-$	4.46	5.16E-06	1.43
Glutamine	145.0609	145.0608	0.77	$[C_5H_{10}N_2O-H]^-$	1.94	2.06E-03	1.28
Glutamic acid	146.0449	146.0448	0.52	$[C_5H_9NO_4-H]^-$	2.22	2.25E-06	1.69
Phenylalanine	164.0707	164.0706	0.27	$[C_9H_{11}NO_2-H]^-$	3.14	2.74E-08	1.12
Citrulline	174.0874	174.0873	0.24	$[C_6H_{13}N_3O_3-H]^-$	-8.82	2.79E-08	1.99
Glucose	179.0550	179.0550	0.03	$[C_6H_{12}O_6-H]^-$	3.39	1.79E-04	1.12
Hippuric acid	180.0657	180.0655	1.11	$[C_9H_9NO_3+H]^+$	-2.77	2.75E-05	1.36
N-Acetyl-L-glutamic acid	188.0554	188.0553	0.06	$[C_7H_{11}NO_5-H]^-$	1.57	3.39E-04	1.27
N8-Acetylspermidine	188.1758	188.1757	0.54	$[C_9H_{21}N_3O+H]^+$	-2.14	1.53E-03	1.07
N-Acetyl-L-methionine	192.0692	192.0689	1.61	$[C_7H_{13}NO_3S+H]^+$	2.71	9.18E-06	1.40
Tryptophan	203.0820	203.0815	2.44	$[C_{11}H_{12}N_2O_2-H]^-$	1.42	5.03E-04	1.06
Leucylproline	229.1549	229.1547	0.96	$[C_{11}H_{20}N_2O_3+H]^+$	2.49	2.06E-06	1.28
N-Acetyltryptophan	245.0927	245.0921	2.58	$[C_{13}H_{14}N_2O_3-H]^-$	7.65	1.11E-07	1.80
Inosine	267.0737	267.0724	4.88	$[C_{10}H_{12}N_4O_5-H]^-$	2.87	2.23E-04	1.02
Vaccenic acid	281.2486	281.2475	3.99	$[C_{18}H_{34}O_2-H]^-$	-3.61	2.34E-05	1.18
Guanosine	282.0839	282.0833	2.00	$[C_{10}H_{13}N_5O_5-H]^-$	4.07	3.43E-06	1.26
	284.0985	284.0989	-1.64	$[C_{10}H_{13}N_5O_5+H]^+$	3.90	1.96E-03	1.90
Xanthosine	283.0682	283.0673	2.97	$[C_{10}H_{12}N_4O_6-H]^-$	3.37	2.30E-05	1.16
Glutathione	306.0756	306.0754	0.51	$[C_{10}H_{17}N_3O_6S-H]^-$	2.62	3.19E-04	1.33
Adenosine monophosphate	346.0549	346.0547	0.46	$[C_{10}H_{14}N_5O_7P-H]^-$	4.62	1.25E-06	1.33
	348.0700	348.0704	-0.92	$[C_{10}H_{14}N_5O_7P+H]^+$	2.74	9.57E-06	1.33
	370.0522	370.0523	-0.42	$[C_{10}H_{14}N_5O_7P+Na]^+$	4.23	9.43E-07	1.76
Riboflavin	375.1296	375.1299	-0.72	$[C_{17}H_{20}N_4O_6-H]^-$	3.78	7.92E-08	1.28
7-Ketocholesterol	401.3407	401.3414	-1.69	$[C_{27}H_{44}O_2+H]^+$	5.95	7.48E-09	2.20
LPE 16:0	452.2785	452.2772	2.95	$[C_{21}H_{44}NO_7P-H]^-$	-3.07	4.11E-06	1.17
LPE 18:3	476.2780	476.2772	1.79	$[C_{23}H_{42}NO_7P+H]^+$	-1.63	9.75E-03	1.10
LPE 18:2	478.2932	478.2928	0.70	$[C_{23}H_{44}NO_7P+H]^+$	-3.40	1.82E-04	1.19
LPE 18:1	478.2932	478.2928	0.76	$[C_{23}H_{46}NO_7P-H]^-$	-2.50	8.31E-07	1.03
	480.3096	480.3085	2.30	$[C_{23}H_{46}NO_7P+H]^+$	-3.31	3.02E-05	1.22
LPC 16:0	496.3396	496.3398	-0.39	$[C_{24}H_{50}NO_7P+H]^+$	-3.93	4.08E-05	1.43
LPC 18:1	522.3570	522.3554	3.01	$[C_{26}H_{52}NO_7P+H]^+$	-4.19	2.13E-05	1.43
Oxidized glutathione	611.1447	611.1436	1.84	$[C_{20}H_{32}N_6O_{12}S_2-H]^-$	1.65	1.40E-03	1.21
Acetyl-CoA	808.1207	808.1174	4.05	$[C_{23}H_{38}N_7O_{17}P_3S-H]^-$	-6.05	5.62E-03	1.44

Supplementary Table 2. Optimization of MS parameters of 13 target metabolites. DP declustering potential, CE collision of energy.

Target metabolite	Q1 (m/z)	Q3 (m/z)	Dwell time (ms)	DP (V)	CE (V)
Lactic acid	89.0	43.0	5	-80	-13.96
	89.0	45.0	5	-80	-14.24
Succinic acid	116.9	73.0	5	-80	-14.90
	116.9	99.2	5	-80	-15.99
Pyroglutamic acid	128.0	82.2	5	-80	-15.61
	128.0	84.0	5	-80	-14.54
Aspartic acid	132.0	88.1	5	-80	-16.02
	132.0	115.0	5	-80	-15.43
Malic acid	133.0	72.9	5	-80	-23.10
	133.0	114.9	5	-80	-12.83
Glutamine	145.0	109.0	5	-80	-15.26
	145.0	126.9	5	-80	-14.98
Glutamic acid	145.8	102.0	5	-80	-18.09
	145.8	127.9	5	-80	-12.98
Phenylalanine	164.1	102.9	5	-80	-20.86
	164.1	147.1	5	-80	-14.87
Glucose	179.0	58.9	5	-80	-15.03
	179.0	70.9	5	-80	-14.97
N-Acetyl-L-glutamic acid	188.0	102.0	5	-80	-22.86
	188.0	128.0	5	-80	-17.20
Tryptophan	203.0	74.0	5	-80	-20.42
	203.0	116.0	5	-80	-23.37
Glutathione	306.4	127.9	5	-80	-37.33
	306.4	142.8	5	-80	-27.05
Oxidized glutathione	305.2	272.0	5	-80	-15.12
	305.2	143.0	5	-80	-23.83

Supplementary Table 3. The parameters of the single-cell quantitative mass spectrometry platform for quantification of the target metabolites. The parameters include linear range, LOD, LLOQ, accuracy and precision. LOD limit of detection, LLOQ lower limit of quantification, QC quality control, CV coefficient of variation.

Metabolite	Linear range (mM)	R^2	LOD (mM)	LLOQ (mM)	%Bias	QC (LLOQ)	
						Intraday precision (%CV)	Interday precision (%CV)
Lactic acid	0.500 - 10.0	0.9727	1.18×10^{-1}	5.00×10^{-1}	-0.7	8.2	8.2
Succinic acid	0.025 - 0.500	0.9803	6.53×10^{-3}	2.50×10^{-2}	-2.9	13.8	13.2
Pyroglutamic acid	0.010 - 0.500	0.9663	3.23×10^{-3}	1.00×10^{-2}	5.9	9.9	10.3
Aspartic acid	0.500 - 10.0	0.9899	1.47×10^{-1}	5.00×10^{-1}	1.9	10.4	10.3
Malic acid	0.500 - 10.0	0.9680	1.53×10^{-1}	5.00×10^{-1}	-4.7	11.0	10.6
Glutamine	0.025 - 0.400	0.9647	7.21×10^{-3}	2.50×10^{-2}	6.4	13.0	12.2
Glutamic acid	0.500 - 10.0	0.9813	9.51×10^{-2}	5.00×10^{-1}	4.7	8.4	8.2
Phenylalanine	0.050 - 1.00	0.9907	1.38×10^{-2}	5.00×10^{-2}	2.0	11.7	12.0
N-Acetyl-L-glutamic acid	0.001 - 0.050	0.9750	3.07×10^{-4}	1.00×10^{-3}	13.3	18.1	17.5
Tryptophan	0.025 - 0.500	0.9493	7.09×10^{-3}	2.50×10^{-2}	-4.5	17.0	16.2
Glutathione	0.100 - 10.0	0.9758	3.16×10^{-2}	1.00×10^{-1}	-7.8	16.2	15.4

Supplementary Table 4. Clinical characteristics and biomarker levels of colorectal cancer patients in the training cohort and the test cohort. CEA carcinoembryonic antigen, CA 19-9 carbohydrate antigen 19-9.

	Training cohort (n=60)	Test cohort (n=15)
**Age, years**		
Median	57	56
Range	32-80	32-75
**Sex**		
Male	39 (65.0%)	7 (46.7%)
Female	21 (35.0%)	8 (53.3%)
**Duke**		
A	36 (60.0%)	8 (53.3%)
B	24 (40.0%)	7 (46.7%)
**Grade of differentiation**		
Well	5 (8.3%)	2 (13.3%)
Moderate	52 (86.7%)	12 (80.0%)
Poor	3 (5.0%)	1 (6.7%)
**Metastasis status**		
Non-metastasis	33 (55.0%)	9 (60.0%)
Lymphatic vessel invasion	20 (33.3%)	5 (33.3%)
Distant invasion	7 (11.7%)	1 (6.7%)
**CEA (ng/mL)**		
≤ 3.5	28 (46.7%)	6 (40.0%)
> 3.5	32 (53.3%)	9 (60.0%)
**CA19-9 (U/mL)**		
≤ 37.0	52 (86.7%)	13 (86.7%)
> 37.0	8 (13.3%)	2 (13.3%)

Supplementary Table 5. Total CTC count, C1 CTC count and C2 CTC count of each patient in the training cohort.

Patient ID	Metastasis status	Total CTC count	Classified CTC count	
			C1 subgroup	C2 subgroup
1	non-metastasis	3	2	1
2	non-metastasis	3	2	1
3	non-metastasis	3	2	1
4	non-metastasis	3	3	0
5	non-metastasis	5	4	1
6	non-metastasis	3	2	1
7	non-metastasis	4	3	1
8	non-metastasis	3	3	0
9	non-metastasis	2	2	0
10	non-metastasis	1	1	0
11	non-metastasis	3	2	1
12	non-metastasis	3	2	1
13	non-metastasis	5	1	4
14	non-metastasis	4	3	1
15	non-metastasis	3	3	0
16	non-metastasis	4	3	1
17	non-metastasis	2	2	0
18	non-metastasis	3	2	1
19	non-metastasis	3	3	0
20	non-metastasis	4	3	1
21	non-metastasis	5	4	1
22	non-metastasis	2	2	0
23	non-metastasis	5	3	2
24	non-metastasis	5	4	1
25	non-metastasis	3	3	0
26	non-metastasis	3	1	2
27	non-metastasis	4	4	0
28	non-metastasis	5	2	3
29	non-metastasis	3	2	1
30	non-metastasis	3	2	1
31	non-metastasis	5	3	2
32	non-metastasis	4	2	2
33	non-metastasis	5	2	3
34	metastasis	3	1	2
35	metastasis	4	1	3
36	metastasis	2	0	2
37	metastasis	1	0	1
38	metastasis	4	2	2
39	metastasis	6	2	4
40	metastasis	3	0	3
41	metastasis	6	1	5
42	metastasis	5	2	3
43	metastasis	4	0	4
44	metastasis	4	1	3
45	metastasis	3	0	3
46	metastasis	5	0	5
47	metastasis	5	2	3
48	metastasis	4	0	4
49	metastasis	5	0	5
50	metastasis	5	2	3
51	metastasis	4	2	2
52	metastasis	5	2	3
53	metastasis	4	0	4
54	metastasis	5	0	5
55	metastasis	4	0	4
56	metastasis	4	0	4
57	metastasis	5	2	3
58	metastasis	5	2	3
59	metastasis	4	1	3
60	metastasis	4	2	2

Supplementary Table 6. Metastasis status of 60 colorectal cancer patients and the corresponding results of SVM-based machine learning method in the training cohort. Accuracy, sensitivity and specificity of the SVM method were 84.3%, 81.8% and 87.0% for CTCs, respectively.

Patient ID	Metastasis status	Total CTC count	Classified CTC count		CTC Heterogeneity (%)
			Non-metastasis	Metastasis	
1	non-metastasis	3	3	0	0.0
2	non-metastasis	3	2	1	33.3
3	non-metastasis	3	3	0	0.0
4	non-metastasis	3	3	0	0.0
5	non-metastasis	5	5	0	0.0
6	non-metastasis	3	3	0	0.0
7	non-metastasis	4	2	2	50.0
8	non-metastasis	3	3	0	0.0
9	non-metastasis	2	2	0	0.0
10	non-metastasis	1	1	0	0.0
11	non-metastasis	3	2	1	33.3
12	non-metastasis	3	3	0	0.0
13	non-metastasis	5	4	1	20.0
14	non-metastasis	4	2	2	50.0
15	non-metastasis	3	2	1	33.3
16	non-metastasis	4	3	1	25.0
17	non-metastasis	2	2	0	0.0
18	non-metastasis	3	3	0	0.0
19	non-metastasis	3	3	0	0.0
20	non-metastasis	4	3	1	25.0
21	non-metastasis	5	4	1	20.0
22	non-metastasis	2	2	0	0.0
23	non-metastasis	5	4	1	20.0
24	non-metastasis	5	5	0	0.0
25	non-metastasis	3	3	0	0.0
26	non-metastasis	3	2	1	33.3
27	non-metastasis	4	2	2	50.0
28	non-metastasis	5	4	1	20.0
29	non-metastasis	3	1	2	66.7
30	non-metastasis	3	2	1	33.3
31	non-metastasis	5	5	0	0.0
32	non-metastasis	4	2	2	50.0
33	non-metastasis	5	4	1	20.0
34	metastasis	3	0	3	0.0
35	metastasis	4	0	4	0.0
36	metastasis	2	0	2	0.0
37	metastasis	1	0	1	0.0
38	metastasis	4	0	4	0.0
39	metastasis	6	3	3	50.0
40	metastasis	3	0	3	0.0
41	metastasis	6	1	5	16.7
42	metastasis	5	2	3	40.0
43	metastasis	4	0	4	0.0
44	metastasis	4	1	3	25.0
45	metastasis	3	0	3	0.0
46	metastasis	5	1	4	20.0
47	metastasis	5	1	4	20.0
48	metastasis	4	0	4	0.0
49	metastasis	5	0	5	0.0
50	metastasis	5	1	4	20.0
51	metastasis	4	0	4	0.0
52	metastasis	5	1	4	20.0
53	metastasis	4	0	4	0.0
54	metastasis	5	0	5	0.0
55	metastasis	4	0	4	0.0
56	metastasis	4	1	3	25.0
57	metastasis	5	0	5	0.0
58	metastasis	5	1	4	20.0
59	metastasis	4	0	4	0.0
60	metastasis	4	1	3	25.0

Supplementary Table 7. Validation of SVM-based method in the test cohort. Accuracy, sensitivity and specificity of the SVM method were 69.8%, 69.2% and 70.4% for CTCs, respectively. Only 37 out of 53 CTCs (69.8%) in 15 patients were classified correctly, in which disagreement with the theoretical ones was observed in 13/15 patients.

Patient ID	Metastasis status	Total CTC count	Classified CTC count		CTC Heterogeneity (%)
			Non-metastasis	Metastasis	
1	non-metastasis	3	3	0	0.0
2	non-metastasis	3	2	1	33.3
3	non-metastasis	2	1	1	50.0
4	non-metastasis	3	3	0	0.0
5	non-metastasis	2	1	1	50.0
6	non-metastasis	3	2	1	33.3
7	non-metastasis	3	2	1	33.3
8	non-metastasis	5	3	2	40.0
9	non-metastasis	3	2	1	33.3
10	metastasis	2	0	2	0.0
11	metastasis	5	3	2	60.0
12	metastasis	3	1	2	33.3
13	metastasis	5	2	3	40.0
14	metastasis	6	1	5	16.7
15	metastasis	5	1	4	20.0

Supplementary Table 8. Univariate and multivariate logistic regression analyses of CTC counts, clinical characteristics and biomarker levels with metastatic risk of colorectal cancer in the training cohort. Statistical analysis was performed using the logistic regression models. CEA carcinoembryonic antigen, CA 19-9 carbohydrate antigen 19-9.

	Non-metastasis	Metastasis	P	
			Univariate	Multivariate
Age, years				
≤ 60	17	16	0.549	-
> 60	16	11		
Sex				
male	23	16	0.400	-
female	10	11		
Duke				
A	23	13	0.093	0.224
B	10	14		
CEA (ng/mL)				
≤ 3.5	17	11	0.406	-
> 3.5	16	16		
CA 19-9 (U/mL)				
≤ 37.0	31	21	0.085	0.223
> 37.0	2	6		
Total CTC count				
≤ 3	19	5	0.003	0.440
> 3	14	22		
C1 CTC count				
> 1	30	10	< 0.001	0.074
≤ 1	3	17		
C2 CTC count				
≤ 1	26	1	< 0.001	0.015
> 1	7	26		

Supplementary Table 9. Clinical information of 5 CTC-positive colorectal cancer patients in the prospective cohort. CEA carcinoembryonic antigen, CA 19-9 carbohydrate antigen 19-9.

Patient ID	Age	Sex	Total CTC count	C1 CTC count	C2 CTC count	CEA (ng/mL)	CA19-9 (U/mL)
1	56	Female	4	3	1	2.7	36.3
2	68	Female	4	3	1	1.6	11.1
3	57	Male	3	3	0	1.9	13.9
4	37	Female	5	0	5	9.9	11.8
5	50	Female	3	0	3	2.8	13.6

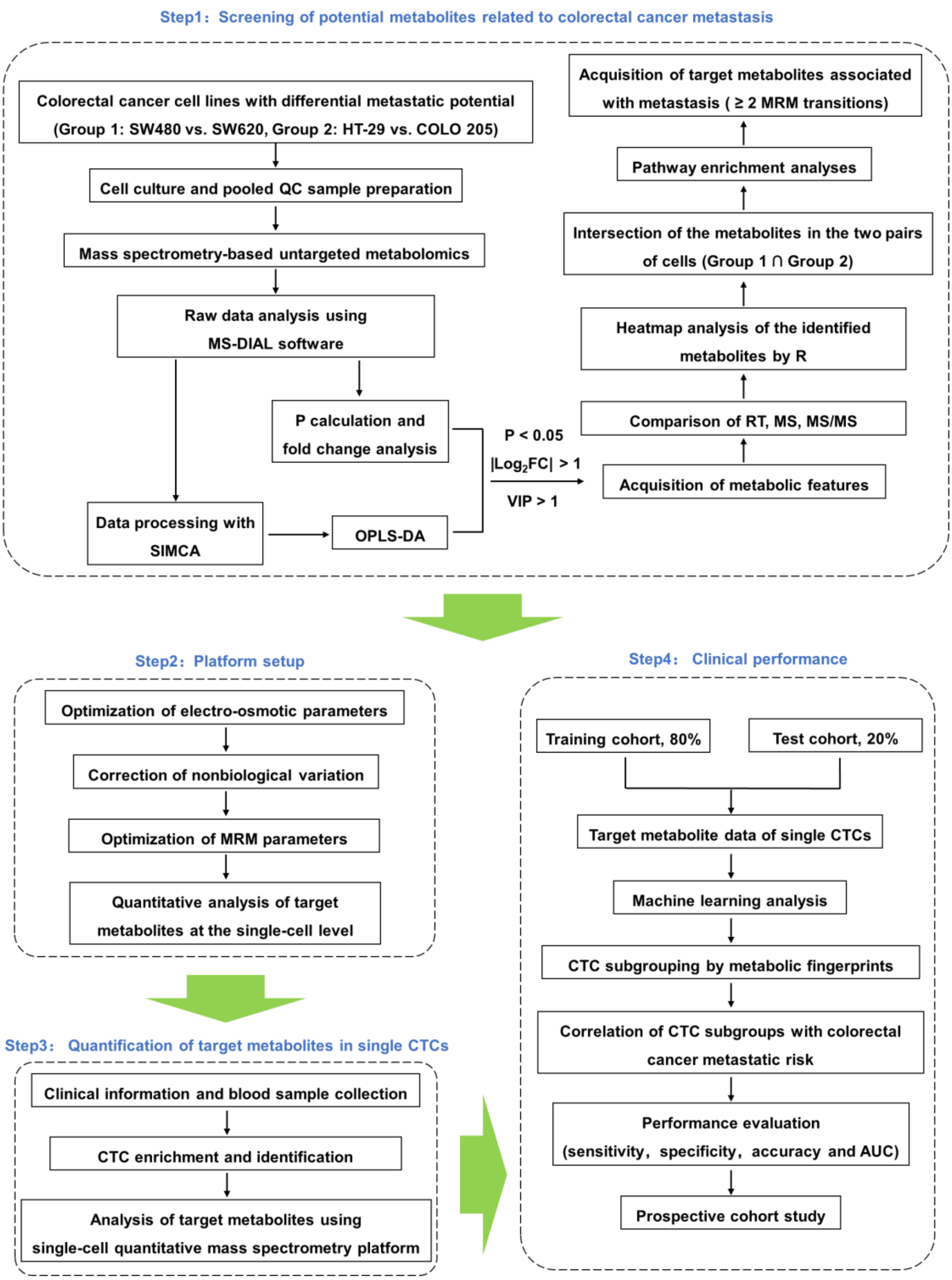

Supplementary Fig. 1. Experimental design of the study. Step 1: Screening of potential metabolites related to colorectal cancer metastasis. Step 2: Setup of home-built single-cell quantitative mass spectrometry platform. Step 3: Quantification of target metabolites in single CTCs. Step 4: CTC subgrouping by metabolic fingerprinting and correlation of subgroups with colorectal cancer metastatic risk. QC quality control, OPLS-DA orthogonal partial least-squares

discriminant analysis, FC fold change, VIP variable important in projection, RT retention time, MRM multiple reaction monitoring, AUC area under curve.

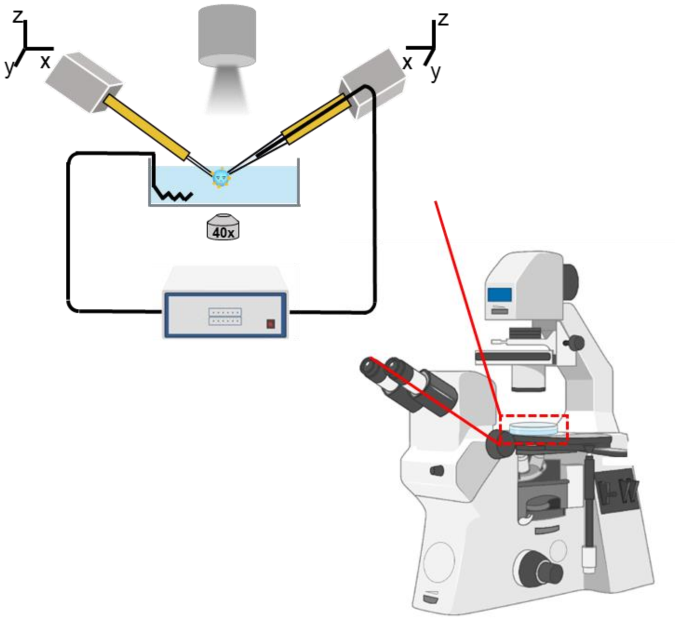

Supplementary Fig. 2. Single-cell micro-sampling. Single CTCs were held with a microcapillary holder and sampled with a pulled nanocapillary, which were held by micromanipulators mounted on an inverted microscope. An Ag/AgCl wire was inserted into the tip of the nanocapillary for electro-osmotic extraction of cellular contents. Some graphical elements were created with BioRender.com (accessed on 24 March 2023).

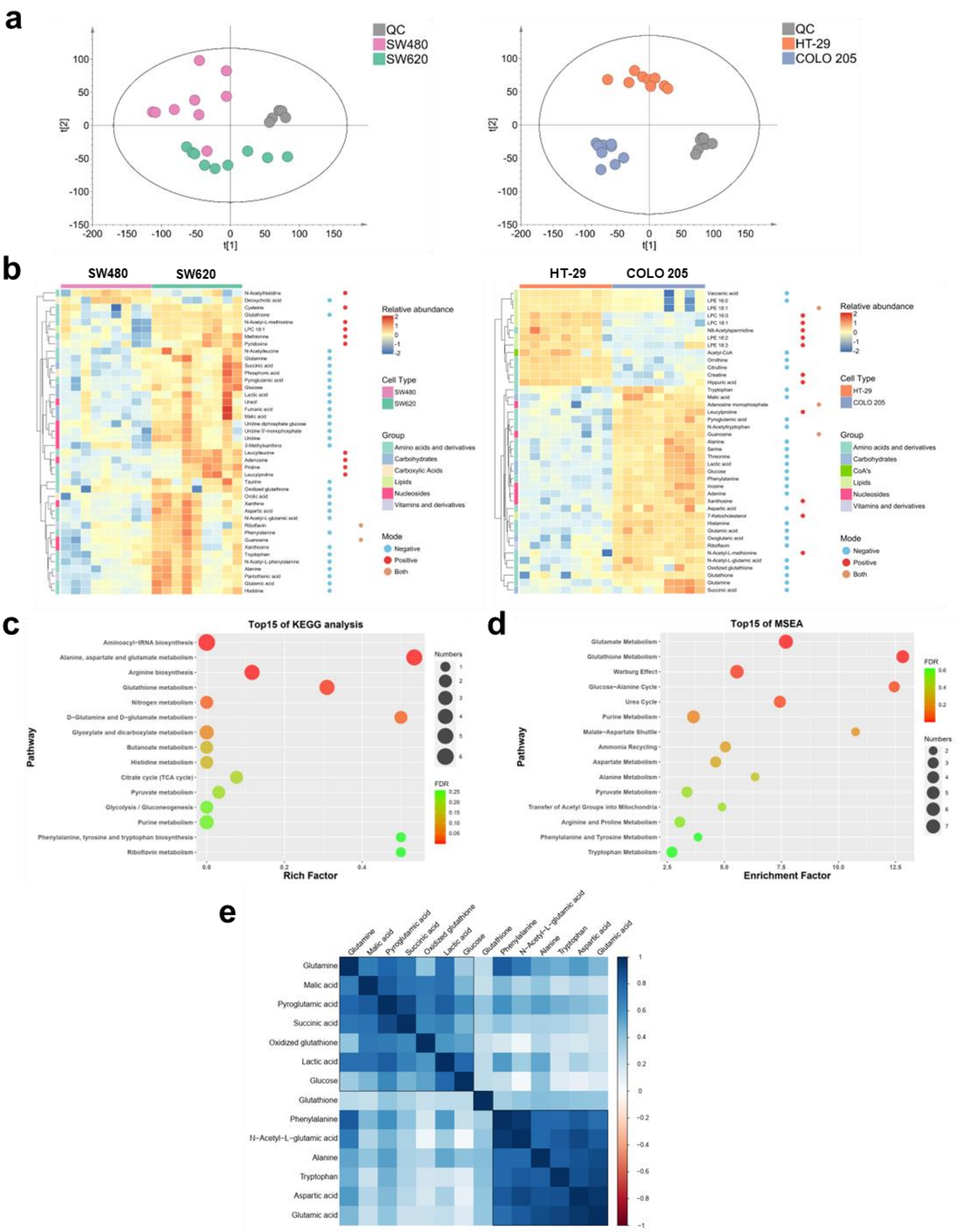

Supplementary Fig. 3. Results of mass spectrometry-based untargeted metabolomic analysis. a PCA results obtained in SW480/SW620 cells (left) and HT-29/COLO 205 cells (right). **b** Heatmaps of the relative abundance (Log_{10} transformation) of the metabolites with differential abundance in SW480/SW620 cells (left) and HT-29/COLO 205 cells (right). **c** KEGG pathway analysis and **d** MSEA of metabolic pathways by comparing colorectal cancer cells with differential metastatic potential. **e** Correlation analysis of the shared

metabolites with differential abundance. QC quality control, KEGG Kyoto Encyclopedia of Genes and Genomes, MSEA metabolite set enrichment analysis.

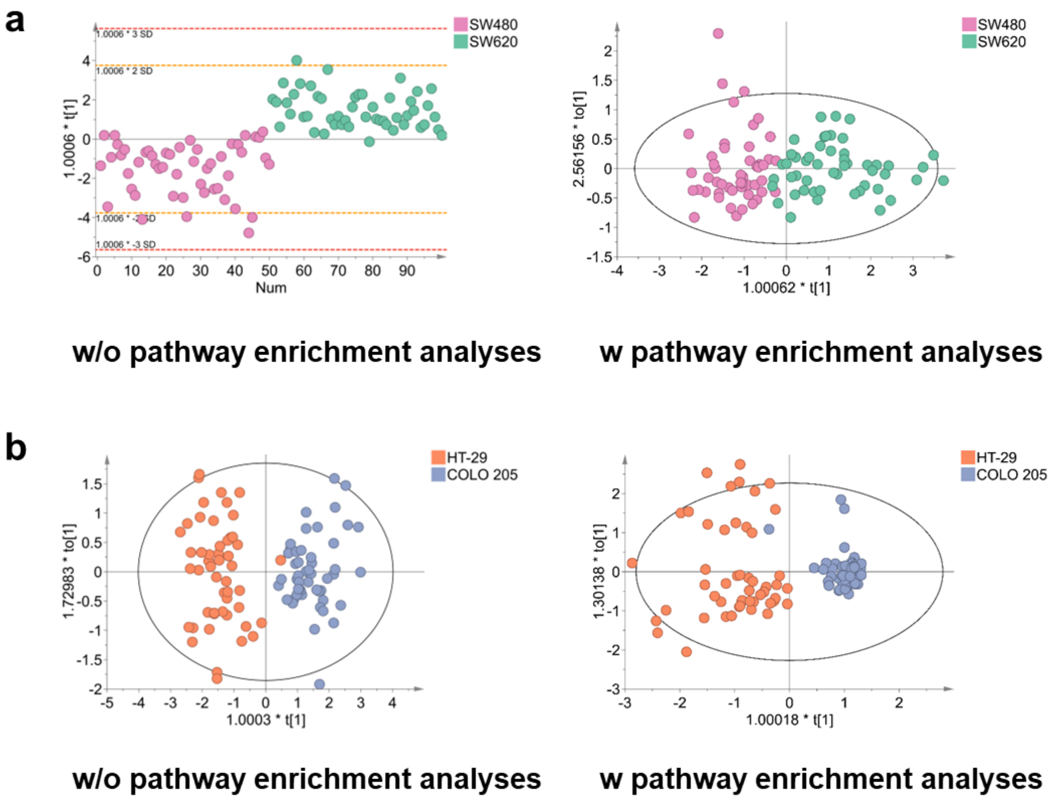

Supplementary Fig. 4. OPLS-DA plots of single cells. OPLS-DA results of **a** SW480/SW620 and **b** HT-29/COLO 205 using 19 metabolites before pathway enrichment analyses (left) and the ultimate 11 metabolites after pathway enrichment analyses (right) are shown. The results demonstrated that pathway enrichment analyses as a knowledge-based dimensionality reduction tool did not oversimplify the dataset in this study. w/o without, w with.

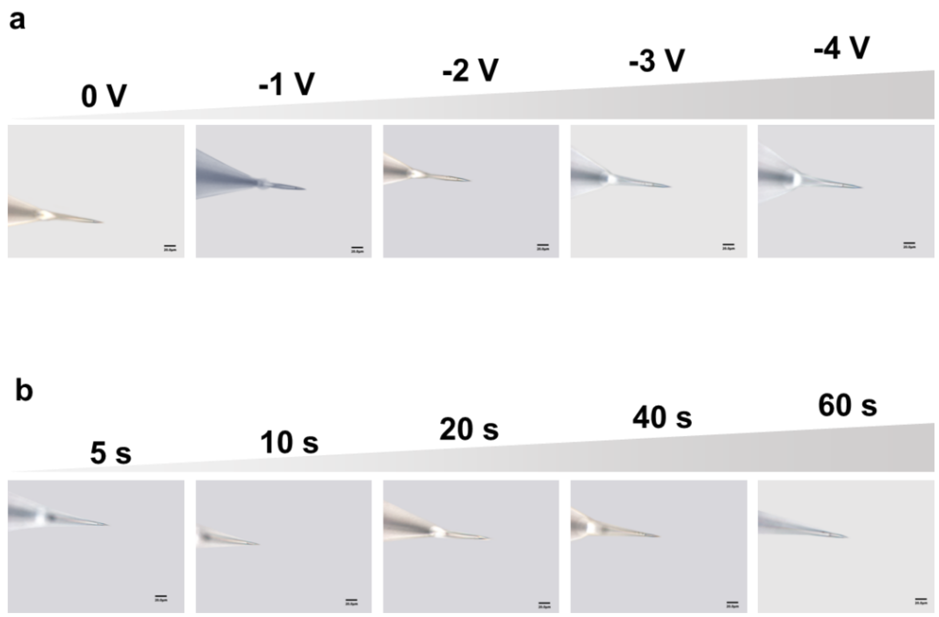

Supplementary Fig. 5. Photographs of the nanocapillary tips during micro-sampling. The images at various extraction **a** voltages (fixed extraction time 40 s) and **b** times (fixed extraction voltage -2 V) are shown, corresponding to **Fig. 3b**. Each experimental condition was repeated three times independently with similar results.

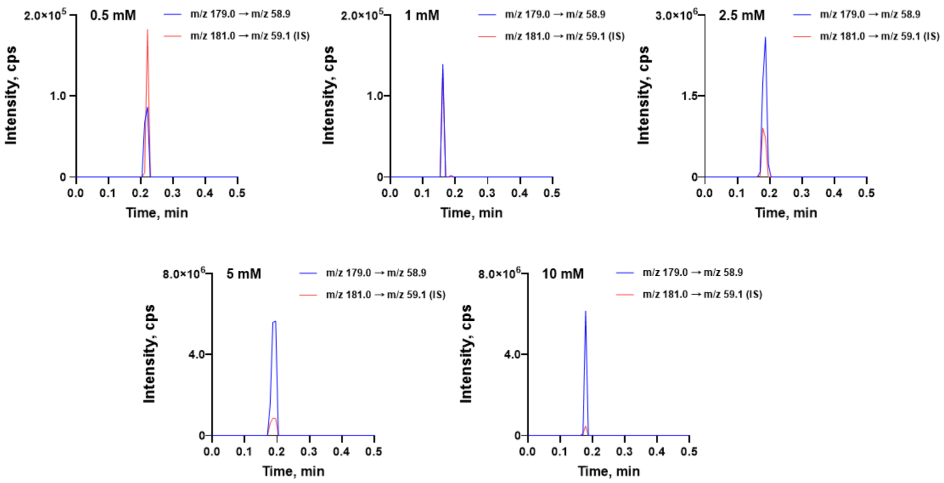

Supplementary Fig. 6. MS intensities of glucose and glucose-d$_2$. Glucose was tested at different concentrations, while glucose-d$_2$ was used as the internal standard. Only one MRM transition of each analyte is shown for clarity. The signals of glucose and glucose-d$_2$ were integrated and used to generate the calibration curve shown in **Fig. 3c**.

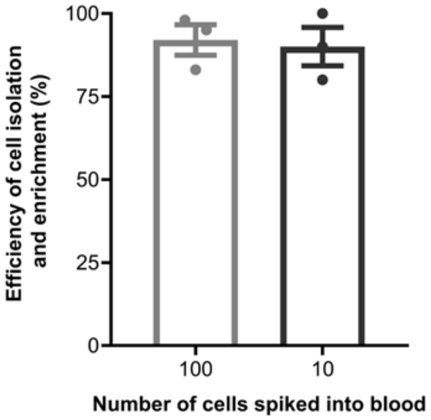

Supplementary Fig. 7. Efficiency of cell isolation by MACS technique. 10 and 100 CFDA-SE labeled colorectal cancer SW480 cells were spiked into 15 ml of fresh blood from a healthy donor respectively, and subjected to MACS. The number of CFDA-SE labeled cells with and without MACS was estimated by using a cytocentrifuge. Data are presented as mean ± SEM. n = 3 independent experiments. The percentage of CFDA-SE labeled cells recovered after MACS is indicated, confirming the efficiency of cell isolation by MACS technique.

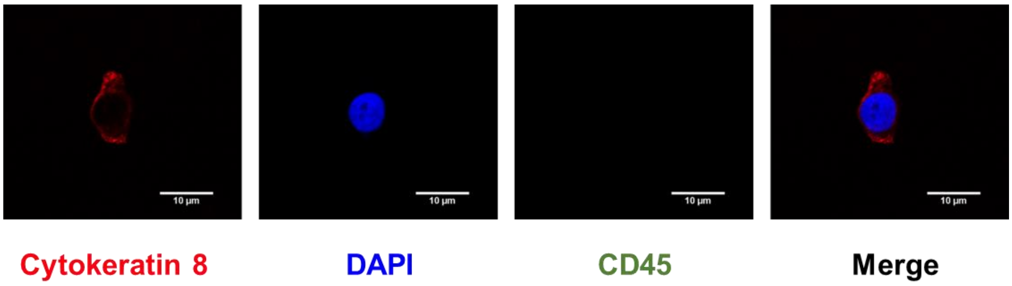

Supplementary Fig. 8. Fluorescent images of single CTC. Cells were immunofluorescence stained and CTCs were defined as CK^{+} (red), $CD45^{-}$ (green) and $DAPI^{+}$ (blue). Experiments were repeated six times independently with similar results.

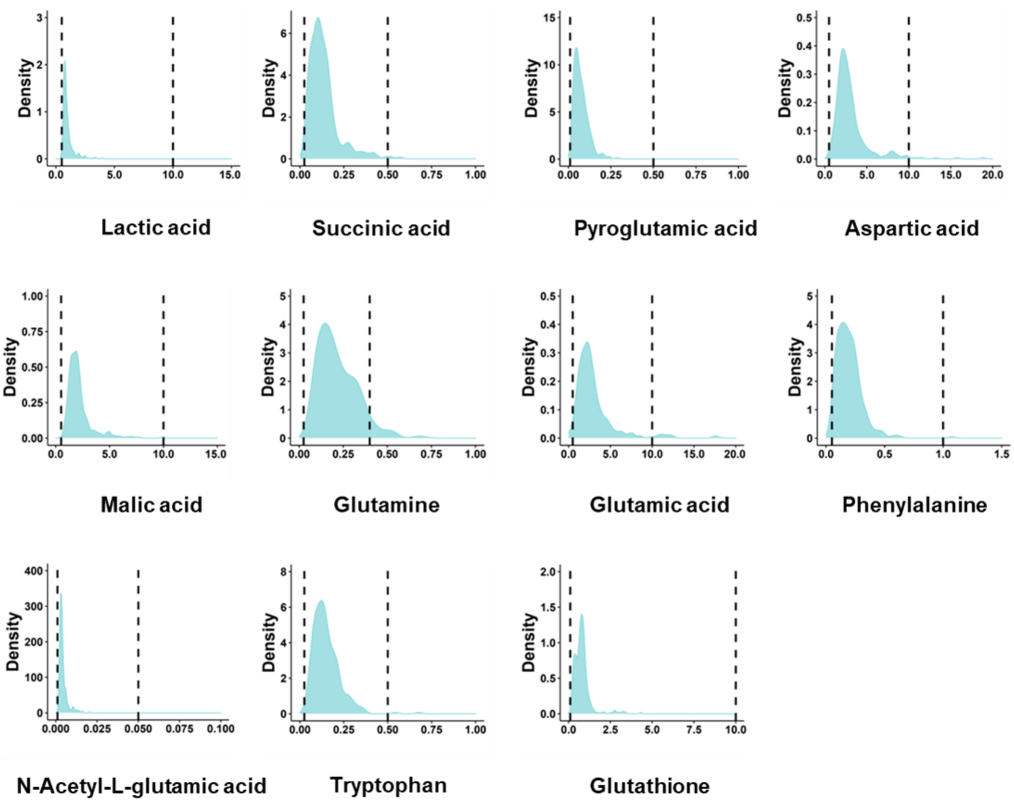

Supplementary Fig. 9. The distributions of CTCs corresponding to the concentration of each target metabolite in CTCs from the patients in the training and test cohorts. The portion of CTCs with the concentration within the range of calibration curve is present between two vertical dashed lines. Nearly 1.37% of the values were outside the concentration range of calibration curves and were extrapolated.

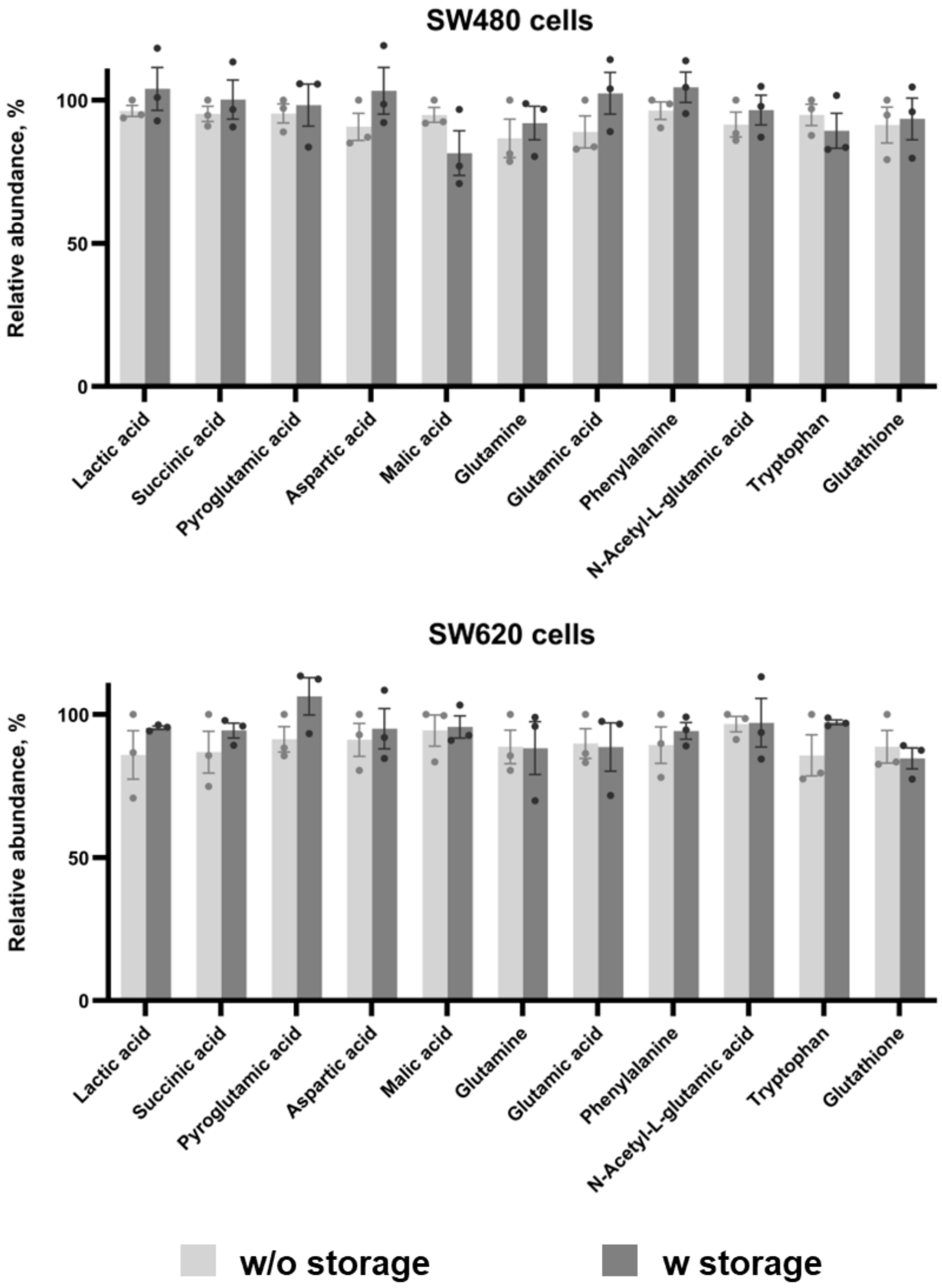

Supplementary Fig. 10. Abundance of the target metabolites in cancer cells spiked in blood after 2 h storage. 1×10^6 SW480/SW620 cells were spiked into 15 ml fresh blood from healthy donors and stored at 4 °C for 2 h before enrichment. The isolated SW480/SW620 cells were further extracted and 11 target metabolites were subjected to LC-MS/MS detection (n = 3 independent experiments). Direct extraction of the metabolites in SW480/SW620 cells without storage was regarded as the control group (n = 3 independent experiments). There was no significant difference in the

abundance of the target metabolites in the two groups ($P > 0.05$). Results are presented as mean ± SEM. Statistical analyses were carried out using the two-tailed Student’s t-test. w/o without, w with.

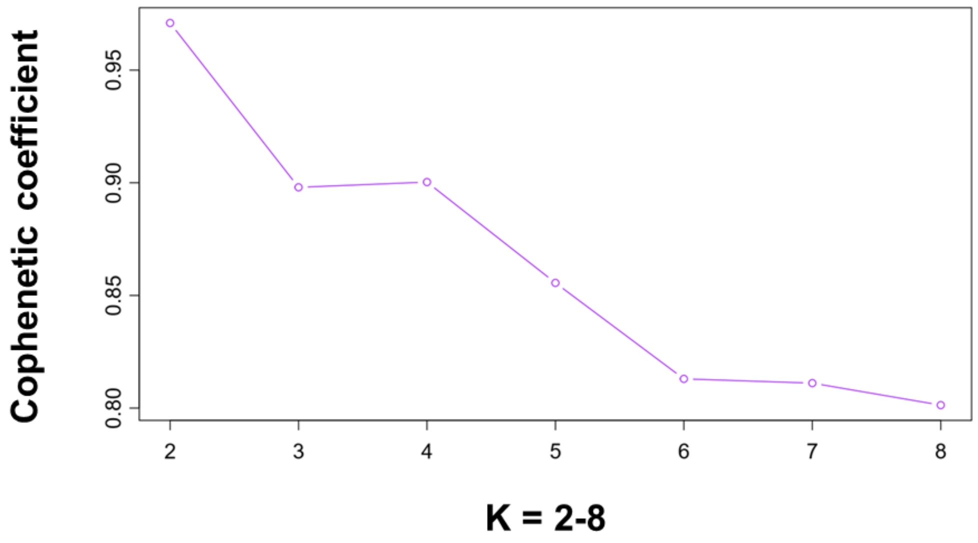

Supplementary Fig. 11. NMF clustering of 11 target metabolites. Cophenetic correlation coefficients for K = 2–8 are shown.

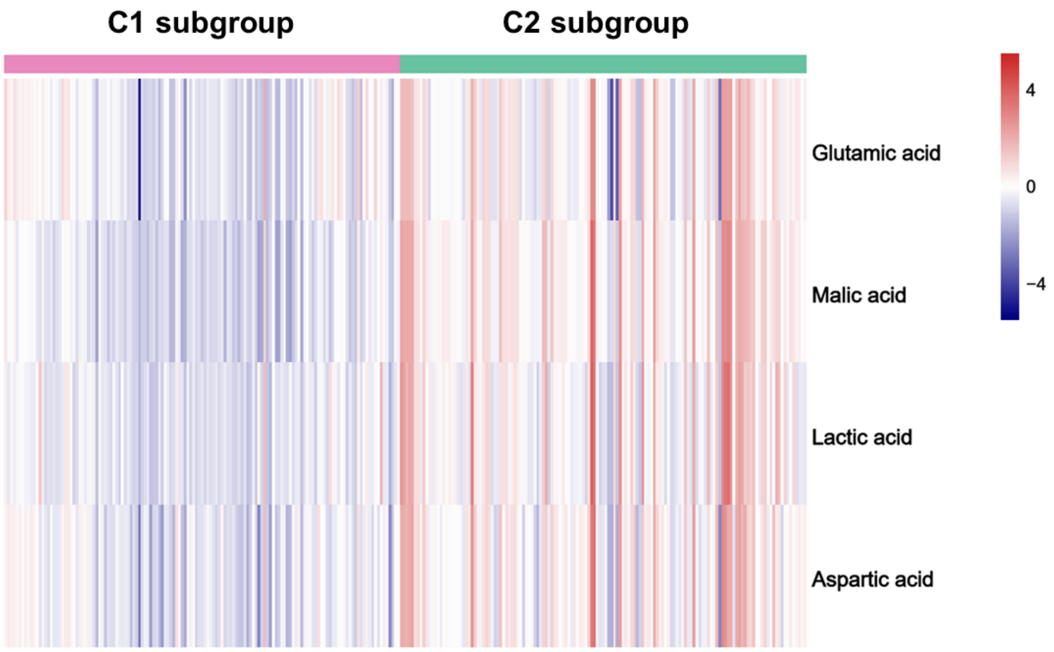

Supplementary Fig. 12. Heatmap of the relative abundance (Log_2 transformation) of the target metabolites with differential abundance in the CTCs of C1 and C2 subgroups.

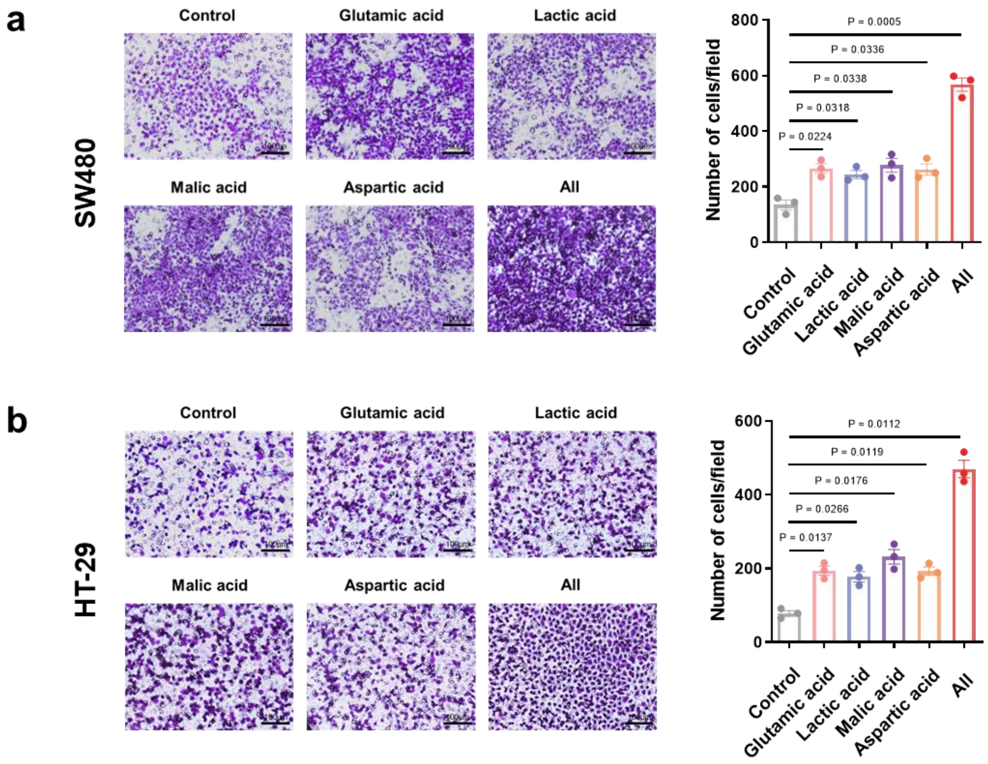

Supplementary Fig. 13. Effect of 4-metabolite fingerprint on the migration of SW480 cells and HT-29 cells. Transwell assay was performed to measure the migratory capacities of **a** SW480 cells and **b** HT-29 cells before and after the addition of 4 target metabolites. Data are expressed as mean ± SEM (n = 3 independent experiments). Statistical analysis was performed using Brown-Forsythe and Welch ANOVA tests followed by Dunnett's T3 test for multiple comparisons. In **a**, glutamic acid group vs. control group, P = 0.0224; lactic acid group vs. control group, P = 0.0318; malic acid group vs. control group, P = 0.0338; aspartic acid group vs. control group, P = 0.0336; all vs. control group, P = 0.0005. In **b**, glutamic acid group vs. control group, P = 0.0137; lactic acid group vs. control group, P = 0.0266; malic acid group vs. control group, P = 0.0176; aspartic acid group vs. control group, P = 0.0119; all vs. control group, P = 0.0112.

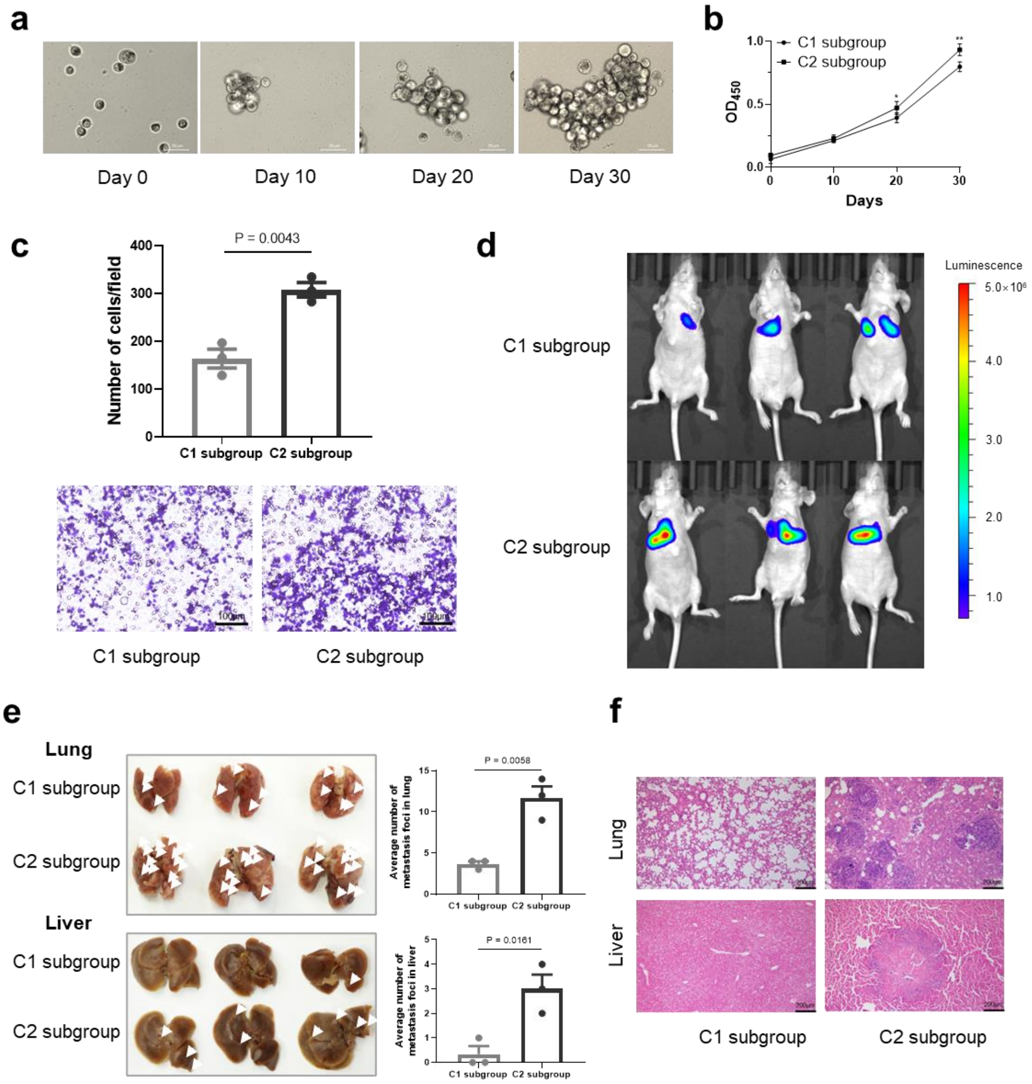

Supplementary Fig. 14. The association of the metabolic phenotype with the metastatic potential of CTC subgroups using CTC-derived model. a Time course images of spheroids formed by cells in C2 subgroup. **b** The growth curves of spheroids formed by cells in two CTC subgroups and detected by CCK-8 assay. **c** Migration of cells in two CTC subgroups. Transwell assay was performed to measure the migratory capacities of cells in C1 and C2 subgroups (n = 3 independent experiments). The number of migrated cells was counted. C2 subgroup vs. C1 subgroup, P = 0.0043. **d** Bioluminescence imaging of CDX model. **e** Representative images of lung and liver tissue samples collected from CDX model (n = 3 independent experiments). Metastatic foci were observed (white arrows). The number of

metastatic foci in lung and liver was counted. Upper, C2 subgroup vs. C1 subgroup, P = 0.0058; Lower, C2 subgroup vs. C1 subgroup, P = 0.0161. **f** Tissue samples were stained with H&E for histological analysis. Values represent mean ± SEM (n = 3 independent experiments). Statistical analysis was performed using the two-tailed Student’s t-test.

Supplementary Method 1. Chemicals and reagents

Metabolite reagents, including glutathione, oxidized glutathione, glutamic acid, glutamine, lactic acid, alanine, malic acid, succinic acid, tryptophan, phenylalanine, aspartic acid, N-acetyl-L-glutamic acid, pyroglutamic acid, glucose, riboflavin, guanosine, xanthosine, N-Acetyl-L-methionine, L-leucyl-L-proline, glucose-6,6-d_2 and 4-acetamidophenol were purchased from Aladdin (Shanghai, China). LC-MS grade acetonitrile, methanol, N-butanol, ammonium acetate and Cell Counting Kit-8 (CCK-8) were obtained from Sigma-Aldrich (St. Louis, USA). Borosilicate glass capillaries (BF100-58-10) were purchased from Sutter Instrument (Novato, USA). SepMate^{TM} kit was bought from STEMCELL Technologies (Vancouver, Canada). Anti-human EpCAM (CD326, Lot #: 130-061-101, 100 μl per 5 × 10^7 cells) microbeads, anti-human CD45-FITC (Lot #: 130-114-648, 1:50 dilution) and LS magnetic columns were purchased from Miltenyi Biotec (Auburn, USA). Anti-human cytokeratin (CK) 8-APC (Lot #: 304005, 1:50 dilution) were purchased from Biolegend (San Diego, USA). All primary antibodies are commercially available and validated by the manufacturers.

Supplementary Method 2. Cell culture

The low-metastatic colorectal cancer cell lines SW480 and HT-29, and the high-metastatic colorectal cancer cell lines SW620 and COLO 205 were obtained from the Cell Resource Center of the Chinese Academy of Medical

Sciences (Shanghai, China). The identity of the cell lines was authenticated with short tandem repeat (STR) profiling (FBI, CODIS) and all the results can be viewed on the website (http://cellresource.cn). In detail, SW480 cells and SW620 cells were cultured in DMEM medium supplemented with 10% FBS at 37 °C under a 5% CO_2 atmosphere, HT-29 and COLO 205 cells were cultured in RPMI 1640 medium supplemented with 10% FBS at 37 °C under a 5% CO_2 atmosphere. Cells were split every 2–3 days by lifting cells with 0.25% trypsin and feeding between splits with the addition of fresh medium. All experiments were performed using the cells in the exponential growth phase. And low-passage colorectal cancer cells were used to minimize genetic drift[1].

Supplementary Method 3. Clinical sample collection

In this study, 225 patients were selected randomly from the hospitalized colorectal cancer patients with no metastasis at Jiangsu Cancer Hospital and Sir Run Run Hospital Affiliated to Nanjing Medical University between January 2018 and August 2021. Blood samples of the patients were collected, among which 208 patients met the inclusion criteria and were enrolled in the training and test cohorts. The other 17 patients were excluded mainly because of a previous history of cancers. More specifically, the inclusion criteria of the study were as follows: (1) ≥18 years old; (2) pathologically diagnosed with colorectal cancer without preoperative chemo- or radiotherapy; (3) no evidence of metastasis including lymph nodes, liver, lung, and peritoneum was found by

imaging or surgery; and (4) absence of other concomitant or previous malignant disease within five years. We then followed these patients for up to two years. During the follow-up, each patient was interviewed or phoned by professional clinician in hospitals every three months and their health information including metastasis occurrence and metastatic site was recorded using a standard questionnaire. Clinical characteristics and biomarker levels, including age, sex, Duke, grade of differentiation, carcinoembryonic antigen (CEA), carbohydrate antigen 19-9 (CA 19-9) and metastasis status, were retrieved. Furthermore, 15 additional colorectal cancer patients were independently enrolled in the prospective study at Jiangsu Cancer Hospital and Sir Run Run Hospital Affiliated to Nanjing Medical University between January 2019 and August 2021 using the same inclusion and exclusion criteria. Informed consent including consent for publishing images was obtained from each patient. This study was approved by the Institutional Review Board of Jiangsu Cancer Hospital and Sir Run Run Hospital Affiliated to Nanjing Medical University, Nanjing, China.

Supplementary Method 4. CTC enrichment and identification

Approximately 15 ml of blood was collected in tubes that contain ethylenediaminetetraacetic acid (EDTA) to prevent clotting[2]. Afterward, CTCs in blood were magnetically enriched by magnetic separation and identified by confocal microscopy using a cytokeratin antibody[3]. Briefly, blood samples were

collected and pretreated with red blood cell (RBC) lysis buffer to remove erythrocytes. Erythrocytes were lysed by adding 6 volumes of 1× RBC lysis buffer, mixing by inverting, and incubating for 5 min at room temperature. Lysed blood was then centrifuged at 500 × g for 5 min. The lysis step was repeated, and the cell pellet was then resuspended in 1 ml of PBS buffer. Samples were centrifuged at 300 × g for 5 min. Finally, the cell pellet was resuspended in 0.9 ml of PBS. Then, 100 μl of EpCAM (CD326) microbeads were added to 0.9 ml of cell suspension. The cell-microbead mixture was incubated for 30 min on a shaker. An LS magnetic column was assembled on a MidiMACS magnet and used for CTC enrichment. The column containing the CTCs was then disassembled from the magnet, and the CTCs were eluted from the column with 3 ml of PBS and collected into a Corning dish for single-cell analysis. The isolated CTCs were stained with an APC-conjugated antibody against cytokeratin 8, a FITC-conjugated antibody to CD45 and the nuclear dye DAPI. Cell images were obtained with an immunoconfocal microscope (LSM 710, Zeiss, Oberkochen, Germany).

Supplementary Method 5. Validation of CTC enrichment

For technical validation of CTC separation and enrichment by magnetic-activated cell sorting (MACS) technique, SW480 cells were labeled with carboxyfluorescein diacetate succinimidyl ester (CFDA-SE) at 2 μM for 10 min and spiked into 15 ml of normal blood before enrichment. After CTC

enrichment, the sample was centrifuged using a Shandon Cytospin 3 cytocentrifuge at 500 rpm for 5 min onto a glass slide. Then, the number of CFDA-SE labeled cells on the slide was counted by two individual counters three times each. The result indicated that there was no significant difference between the numbers of cells with and without MACS.

Supplementary Method 6. Correction of nonbiological variations

Mass spectrometry detection can be sensitive to nonbiological variations, including both technical variability arising through technical effects and confounding factors such as batch effects experienced between biological replicates[4]. In this study, technical variability was corrected using spike-in approach and batch effects were removed using ComBat function. In detail, we incorporated quantitative standards for data normalization to correct the technical variability[5]. We added stable isotope-labeled internal standard of known concentration to nanocapillary using a second electro-osmotic extraction after cellular extraction in single-cell micro-sampling. Quantitative information of the target metabolites in single cells was obtained by integrating the areas in the MRM transitions of target metabolites and internal standard. To remove batch effects, tools developed for batch correction such as ComBat were employed in this study[6].

Supplementary Method 7. Preparation of stock solutions, calibration

standards and quality controls (QCs)

Stock solutions of the target metabolites and internal standard were first prepared in deionized water and stored at -20 °C in amber glass tubes. Internal standard solution (1 mM) was prepared by serially diluting the stock solution. Calibration standards of the metabolites were prepared by serially diluting the stock solutions in the surrogate matrix BSA. The linear ranges of the metabolites are listed in Supplementary Table 3. QCs were prepared and frozen prior to use.

Supplementary Method 8. Validation of the single-cell quantitative mass spectrometry platform

In general, signal to noise (S/N) is the ratio of the analyte signal to the noise measured on a blank. The limit of detection (LOD) can be determined as an S/N of 3:1. The lower limit of quantification (LLOQ) is the lowest calibration standard on the calibration curve where the detection response for the analyte should be at least ten times over the blank. In this study, we used LLOQ as QC to validate the single-cell quantitative mass spectrometry platform.

To estimate accuracy and precision of the method, validation experiments were conducted in three independent runs over several days. Each run included a calibration curve (in duplicate) and 6 QCs per run with total number of 18. The MS signals of target metabolites and internal standard were integrated and used to generate calibration curves. According to the general

validation criteria[7], the precision of QC should be within 20% of the CV (coefficient of variation) while its accuracy should be within 20% of the nominal concentration.

Supplementary Method 9. Sample size estimation

MetaboAnalyst (http://www.metaboanalyst.ca) was used to calculate the sample size in this study. The sample size was estimated for both SW480/SW620 cells and HT-29/COLO 205 cells at a false discovery rate (FDR) threshold of 0.01, based on the abundance of the target metabolites.

Supplementary Method 10. Statistical analysis

We used several machine learning methods, including non-negative matrix factorization (NMF) coupled with logistic regression and support vector machine (SVM), to analyze the target metabolite data in single CTCs. The performance of the methods was evaluated with four metrics: sensitivity, specificity, accuracy and the area under the curve (AUC). NMF coupled with logistic regression was used to define the metastatic potential of single CTCs, and receiver operating characteristic (ROC) curves were plotted to determine the ability of the proposed classification method to predict metastatic risk. Youden's index (sensitivity + specificity - 1) was used to select the best cutoff point from the ROC curves. For continuous variables, two-tailed Student's t-test was used to calculate the significant difference between two groups.

Brown-Forsythe and Welch ANOVA tests followed by Dunnett's T3 test were used for multiple comparisons. Univariate and multivariate logistic regression analyses of the factors associated with metastatic risk were performed and the factors with $P < 0.1$ in the univariate analysis were considered in the multivariate analysis. Statistical analysis was performed with SPSS (version 26) or R (version 4.0.3). Unless indicated, $P < 0.05$ was considered to be statistically significant.

Supplementary Method 11. Transwell assay

SW480 and HT-29 cells were treated with culture medium as a control or the target metabolites (i.e., 25 mM lactic acid, 50 mM aspartic acid, 50 mM glutamic acid, 25 mM malic acid or all of them) for 24 h. Then, the cells were digested, collected and resuspended in serum-free medium. Transwell chambers for migration assay were precoated with Matrigel (BD Biosciences, San Jose, USA). Then, 1×10^5 cells were seeded into the upper chamber, and 500 μl of culture medium supplemented with the same concentration of metabolites as above was added to the lower chamber. After 48 h incubation at 37 °C, the cells remaining on the upper chamber were gently removed with a cotton swab, and the cells adhering to the lower chamber were fixed with 4% paraformaldehyde and stained with 0.1% crystal violet. Images were captured in five random fields under an optical microscope[8]. For the cells in CTC subgroups, the same procedure was carried out.

Supplementary Method 12. Ex vivo CTC culture

Blood samples were obtained from 492 early-stage colorectal cancer patients between August 2021 and April 2022. For each sample, peripheral blood (30 ml) was processed with the SepMateTM kit (STEMCELL Technologies, Vancouver, Canada) for collection of cells following the manufacturer's instructions. The cells were then washed with 2% FBS in PBS and resuspended in cryopreservation solution (10% DMSO, 90% FBS) for cryopreservation. After resuscitation, CTCs were enriched and classified into C1 and C2 subgroups based on the proposed metabolic fingerprint. For each subgroup, no less than 300 cells were pooled together. The cells were incubated under 2% O_2 at 37°C in ultralow attachment 96-well plates (Corning Inc., Corning, USA) in DMEM/F12 (Gibco, Carlsbad, USA) containing 20 g/ml of insulin (Sigma, St. Louis, USA), 1% N_2 complement (Gibco, Carlsbad, USA), 20 ng/ml of epithelial growth factor (R&D System, Minneapolis, USA), 2 mM of L-Glutamine (Gibco, Carlsbad, USA), 10 ng/ml of fibroblast growth factor-2 (R&D System, Minneapolis, USA) and 2% FBS (Gibco, Carlsbad, USA). After 2-3 weeks, the culture medium was switched to RPMI 1640 and the cells were cultured under 5% CO_2.

Supplementary Method 13. Cell Counting Kit-8 (CCK-8) assay

CCK-8 assay (Sigma, St. Louis, USA) was carried out for detection of cell

proliferation according to manufacturer's instructions. In details, cells were seeded in 96-well plates. CCK-8 reagent was added at 10 μl/well at 0, 10, 20, 30 days. After 2.5 h, the optical density at the 450 nm wavelength (OD_{450}) was measured using a microplate reader (Biotek, Winooski, USA).

Supplementary Method 14. Construction of CDX model

Immunodeficient mice were maintained under specific pathogen-free conditions with a 12 h light/12 h dark cycle, at 23 ± 2 °C with humidity of 55 ± 10%, and were fed with a standard mouse chow diet at the Animal Core Facility of Nanjing Medical University. All mice used in this study were BALB/c nude mice (female, six weeks). Female animals were used according to the reported studies[9, 10]. All the animal experiments were conducted according to the ARRIVE guidelines and the animal use protocol was approved by Institutional Animal Care and Use Committee of NMU (IACUC-2212005). The tumor size did not exceed 10% of body weight, which followed the IACUC guideline.

The CDX model was constructed following the published protocols[10, 11]. In brief, ~10^6 cells in each CTC subgroup were transduced with a LUC-encoding lentiviral vector to allow monitoring of tumor growth with bioluminescence imaging, and subsequently injected into tail vein of immunodeficient mice in 50 μl of a 1:1 mixture of Matrigel (BD Biosciences, San Jose, USA) and RPMI 1640 (Gibco, Carlsbad, USA) (n = 3 per subgroup). The mice underwent

bioluminescence imaging 4 weeks after injection with an IVIS imaging system (Perkin Elmer, Waltham, USA). After sacrifice, lung and liver tissues were collected for histological analysis.

Supplementary Method 15. Hematoxylin and eosin (H&E) staining

Tissue samples were then fixed in 4% paraformaldehyde followed by dehydration, paraffin embedding and sectioning. Afterward, the samples were stained with Harris hematoxylin solution for several minutes. Then, the sections were washed in water, differentiated in 1% acid alcohol for 30 s, and counterstained with eosin for 30 s. Images were acquired on an Olympus BX43 microscope (Olympus, Tokyo, Japan).

References

1. Hughes P, Marshall D, Reid Y, Parkes H, Gelber C. The costs of using unauthenticated, over-passaged cell lines: how much more data do we need? Biotechniques 43, 575, 577-578, 581-572 passim (2007).
2. Williams ES, et al. Generation of Prostate Cancer Patient Derived Xenograft Models from Circulating Tumor Cells. J Vis Exp, 53182 (2015).
3. Konigsberg R, et al. Circulating tumor cells in metastatic colorectal cancer: efficacy and feasibility of different enrichment methods. Cancer Lett 293, 117-123 (2010).
4. Stegle O, Teichmann SA, Marioni JC. Computational and analytical challenges in single-cell transcriptomics. Nat Rev Genet 16, 133-145 (2015).
5. Brennecke P, et al. Accounting for technical noise in single-cell RNA-seq experiments. Nat Methods 10, 1093-1095 (2013).
6. Johnson WE, Li C, Rabinovic A. Adjusting batch effects in microarray expression data using empirical Bayes methods. Biostatistics 8, 118-127 (2007).
7. Center for Drug Evaluation and Research. et al. Bioanalytical method validation guidance for industry. 2018. https://www.fda.gov/regulatory-information/search-fda-guidance-documents/bioanalytical-method-validation-guidance-industry.
8. Wu Y, et al. Circular RNA circCORO1C promotes laryngeal squamous cell carcinoma progression by modulating the let-7c-5p/PBX3 axis. Mol Cancer 19, 99 (2020).

9. De Angelis ML, et al. An organoid model of colorectal circulating tumor cells with stem cell features, hybrid EMT state and distinctive therapy response profile. J Exp Clin Cancer Res 41, 86 (2022).
10. Grillet F, et al. Circulating tumour cells from patients with colorectal cancer have cancer stem cell hallmarks in ex vivo culture. Gut 66, 1802-1810 (2017).
11. Cayrefourcq L, et al. Establishment and characterization of a cell line from human circulating colon cancer cells. Cancer Res 75, 892-901 (2015).
