# Supplementary material for: Single-cell metabolic fingerprints discover a cluster of circulating tumor cells with distinct metastatic potential
Source: Nat Commun. 2023 Apr 29;14:2485. doi: 10.1038/s41467-023-38009-3 (PMC10148826; doi:10.1038/s41467-023-38009-3)
Supplement: Supplementary file 2 — Reporting Summary [file 41467_2023_38009_MOESM2_ESM.pdf]

nature portfolio

Corresponding author(s): Yun Chen

Last updated by author(s): Apr 7, 2023

Reporting Summary

Nature Portfolio wishes to improve the reproducibility of the work that we publish. This form provides structure for consistency and transparency in reporting. For further information on Nature Portfolio policies, see our Editorial Policies and the Editorial Policy Checklist.

Statistics

For all statistical analyses, confirm that the following items are present in the figure legend, table legend, main text, or Methods section.

n/a	Confirmed	
☐	☒	The exact sample size (*n*) for each experimental group/condition, given as a discrete number and unit of measurement
☐	☒	A statement on whether measurements were taken from distinct samples or whether the same sample was measured repeatedly
☐	☒	The statistical test(s) used AND whether they are one- or two-sided<br>*Only common tests should be described solely by name; describe more complex techniques in the Methods section.*
☐	☒	A description of all covariates tested
☐	☒	A description of any assumptions or corrections, such as tests of normality and adjustment for multiple comparisons
☐	☒	A full description of the statistical parameters including central tendency (e.g. means) or other basic estimates (e.g. regression coefficient) AND variation (e.g. standard deviation) or associated estimates of uncertainty (e.g. confidence intervals)
☐	☒	For null hypothesis testing, the test statistic (e.g. F, t, r) with confidence intervals, effect sizes, degrees of freedom and P value noted<br>*Give P values as exact values whenever suitable.*
☒	☐	For Bayesian analysis, information on the choice of priors and Markov chain Monte Carlo settings
☒	☐	For hierarchical and complex designs, identification of the appropriate level for tests and full reporting of outcomes
☐	☒	Estimates of effect sizes (e.g. Cohen's *d*, Pearson's *r*), indicating how they were calculated

Our web collection on statistics for biologists contains articles on many of the points above.

Software and code

Policy information about availability of computer code

Data collection	Thermo Fisher Q-Exactive mass spectrometer and Xcalibur v2.0.7 software were used for metabolomics data collection. AB SCIEX QTRAP 5500 MS/MS system and Analyst v1.6.3 software were used for single-cell quantitative mass spectrometry data collection and processing. Cell images were collected with Zeiss LSM 710 confocal microscope. Nanocapillaries were characterized by JSM-7800F scanning electron microscope.
Data analysis	Metabolomics data were processed using MS-DIAL v4.80 software. Metabolites were identified using MS-DIAL internal database, MassBank, METLIN and human metabolome database. PCA and OPLS-DA were conducted with SIMCA 14.1 software. MetaboAnalyst 5.0 (http://www.metaboanalyst.ca) was used to perform KEGG pathway analysis and metabolite set enrichment analysis (MSEA), and estimate sample size. Machine learning algorithms, including non-negative matrix factorization (R package NMF, v0.23.0) coupled with logistic regression (R function glm), support vector machine (R package e1071, v1.7-9) and Combat (R package sva) were used to analyze target metabolite data. Statistical analysis was performed with SPSS (v26) or R (v4.0.3). Graphs were generated using GraphPad Prism v8.3.0 and R v4.0.3.

For manuscripts utilizing custom algorithms or software that are central to the research but not yet described in published literature, software must be made available to editors and reviewers. We strongly encourage code deposition in a community repository (e.g. GitHub). See the Nature Portfolio guidelines for submitting code & software for further information.

Data

Policy information about availability of data

All manuscripts must include a data availability statement. This statement should provide the following information, where applicable:

- Accession codes, unique identifiers, or web links for publicly available datasets
- A description of any restrictions on data availability
- For clinical datasets or third party data, please ensure that the statement adheres to our policy

The summary statistics for the metabolites that displayed significant differences in cells are shown in Supplementary Table 1 and Source Data. The datasets are available in the Metabolomics Workbench database under the accession number ST002341 and are publicly available at https://www.metabolomicsworkbench.org/data/DRCCMetadata.php?Mode=Study&StudyID=ST002341.

Field-specific reporting

Please select the one below that is the best fit for your research. If you are not sure, read the appropriate sections before making your selection.

☒ Life sciences ☐ Behavioural & social sciences ☐ Ecological, evolutionary & environmental sciences

For a reference copy of the document with all sections, see nature.com/documents/nr-reporting-summary-flat.pdf

Life sciences study design

All studies must disclose on these points even when the disclosure is negative.

Sample size	For single-cell quantitative mass spectrometric analysis of blood samples, inclusion criteria were specified. The inclusion criteria were the main limiting factor for the number of patients enrolled in this study. For untargeted metabolomic analysis, nine biological replicates were performed. In addition, the abundance of target metabolites in subgroups provided the rationale for sufficiency of sample size. For metabolite target analysis of cultured cells, MetaboAnalyst was used as an important tool to estimate the sample size for the two pairs of cancer cell lines (SW480 vs. SW620 and HT-29 vs. COLO 205).
Data exclusions	In metabolomic analysis, the metabolic features with |Log2FC|>1.0, P < 0.05, and VIP > 1 were considered significant. The other features were excluded.
Replication	Unless stated, all experiments were replicated at least three times and are represented as mean +/-SEM as appropriate. For the validation of single-cell quantitative mass spectrometry platform, six replicates of QC per day for three consecutive days were conducted. For calibration curves, three replicates were carried out and 95% confidence interval is shown. For untargeted metabolomic analysis, nine biological replicates were performed. For single-cell quantitative mass spectrometric analysis of SW480/SW620 and HT-29/COLO205 cells, fifty cells were analyzed for each cell line. For scanning electron microscopy analysis of nanocapillary, six independent experiments were performed. For the fluorescent analysis of single CTC, experiments were repeated six times independently. For the construction of CTC-derived explants (CDX) model, three mice per subgroup were used.
Randomization	For clinical study, the blood samples were randomly divided into the training and validation cohorts using R package (R function sample).
Blinding	Blinding was not performed in the analysis.

Reporting for specific materials, systems and methods

We require information from authors about some types of materials, experimental systems and methods used in many studies. Here, indicate whether each material, system or method listed is relevant to your study. If you are not sure if a list item applies to your research, read the appropriate section before selecting a response.

Materials & experimental systems

n/a	Involved in the study	
☐	☒	Antibodies
☐	☒	Eukaryotic cell lines
☒	☐	Palaeontology and archaeology
☐	☒	Animals and other organisms
☐	☒	Human research participants
☒	☐	Clinical data
☒	☐	Dual use research of concern

Methods

n/a	Involved in the study	
☒	☐	ChIP-seq
☒	☐	Flow cytometry
☒	☐	MRI-based neuroimaging

Antibodies

Antibodies used	anti-human EpCAM (CD326) microbeads (MACS, Lot#: 130-061-101, 100 µL per 5 × 107 cells)<br>anti-human cytokeratin 8-APC (Biolegend, Lot#: 304005, 1:50 dilution)<br>anti-human CD45-FITC (MACS, Lot#: 130-114-648, 1:50 dilution)
Validation	All primary antibodies are commercially available and validated by the manufacturers.

Eukaryotic cell lines

Policy information about cell lines

Cell line source(s)	Colorectal cancer cell lines SW480, SW620, HT-29 and COLO 205 were obtained from the Cell Resource Center of the Chinese Academy of Medical Sciences, Shanghai, China.
Authentication	The cell lines were authenticated using short tandem repeat (STRs) profiling by the Cell Resource Center of the Chinese Academy of Medical Sciences, Shanghai, China.
Mycoplasma contamination	The cell lines were tested for mycoplasma contamination every two months. Mycoplasma contamination test results were negative.
Commonly misidentified lines<br>(See ICLAC register)	No commonly misidentified cell lines were used.

Animals and other organisms

Policy information about studies involving animals; ARRIVE guidelines recommended for reporting animal research

Laboratory animals	Immunodeficient mice were maintained in a 12-hour dark/light cycle, at a temperature of 23±2°C and a humidity of 55±10%. All mice used in this study were BALB/c nude mice (female, six weeks).
Wild animals	The study did not involve wild animals.
Field-collected samples	The study did not involve samples collected from the field.
Ethics oversight	All the animal experiments were conducted according to the ARRIVE guidelines and the animal use protocol was approved by Institutional Animal Care and Use Committee of NMU (IACUC-2212005).

Note that full information on the approval of the study protocol must also be provided in the manuscript.

Human research participants

Policy information about studies involving human research participants

Population characteristics	Clinical information of the patients, including age, sex, Duke, grade of differentiation, CEA, CA 19-9 and metastasis status, was retrieved. The patients were followed for up to two years. A detailed description of population characteristics is available in Fig. 4a and Supplemental Table 4, 9.
Recruitment	225 patients were selected randomly from the hospitalized colorectal cancer patients with no metastasis at Jiangsu Cancer Hospital and Sir Run Run Hospital Affiliated to Nanjing Medical University between January 2018 and August 2021. Blood samples of the patients were collected, among which 208 patients met the inclusion criteria and were enrolled in the training and test cohorts. The other 17 patients were excluded mainly because of a previous history of cancers. More specifically, the inclusion criteria of the study were as follows: (1) ≥18 years old; (2) pathologically diagnosed with colorectal cancer without preoperative chemo- or radiotherapy; (3) no evidence of metastasis including lymph nodes, liver, lung, and peritoneum was found by imaging or surgery; and (4) absence of other concomitant or previous malignant disease within five years. We then followed these patients for up to two years. During the follow-up, each patient was interviewed or phoned by professional clinician in hospitals every three months and their health information including metastasis occurrence and metastatic site was recorded using a standard questionnaire. Clinical characteristics and biomarker levels, including age, sex, Duke, grade of differentiation, carcinoembryonic antigen (CEA), carbohydrate antigen 19-9 (CA 19-9) and metastasis status, were retrieved. Furthermore, 15 additional colorectal cancer patients were independently enrolled in the prospective study at Jiangsu Cancer Hospital and Sir Run Run Hospital Affiliated to Nanjing Medical University between January 2019 and August 2021 using the same inclusion and exclusion criteria. Informed consent including consent for publishing images was obtained from each patient.
Ethics oversight	This study was approved by the Institutional Review Board of Jiangsu Cancer Hospital and Sir Run Run Hospital Affiliated to Nanjing Medical University, Nanjing, China.

Note that full information on the approval of the study protocol must also be provided in the manuscript.
